# Current State of Fluid Lipid Biomarkers for Personalized Diagnostics and Therapeutics in Schizophrenia Spectrum Disorders and Related Psychoses: A Narrative Review

**DOI:** 10.3389/fpsyt.2022.885904

**Published:** 2022-05-27

**Authors:** Timothy A. Couttas, Beverly Jieu, Cathrin Rohleder, F. Markus Leweke

**Affiliations:** ^1^Brain and Mind Centre, Faculty of Medicine and Health, The University of Sydney, Sydney, NSW, Australia; ^2^Department of Psychiatry and Psychotherapy, Central Institute of Mental Health, Medical Faculty Mannheim, Heidelberg University, Mannheim, Germany

**Keywords:** psychosis, first-episode psychosis (FEP), clinically high risk patients, lipidomics, metabolomics, LC-MS/MS, biomarkers, peripheral tissue

## Abstract

Schizophrenia spectrum disorders (SSD) are traditionally diagnosed and categorized through clinical assessment, owing to their complex heterogeneity and an insufficient understanding of their underlying pathology. However, disease progression and accurate clinical diagnosis become problematic when differentiating shared aspects amongst mental health conditions. Hence, there is a need for widely accessible biomarkers to identify and track the neurobiological and pathophysiological development of mental health conditions, including SSD. High-throughput omics applications involving the use of liquid chromatography-mass spectrometry (LC-MS) are driving a surge in biological data generation, providing systems-level insight into physiological and pathogenic conditions. Lipidomics is an emerging subset of metabolomics, largely underexplored amongst the omics systems. Lipid profiles in the brain are highly enriched with well-established functions, including maintenance, support, and signal transduction of neuronal signaling pathways, making them a prospective and exciting source of biological material for neuropsychiatric research. Importantly, changes in the lipid composition of the brain appear to extend into the periphery, as there is evidence that circulating lipid alterations correlate with alterations of psychiatric condition(s). The relative accessibility of fluid lipids offers a unique source to acquire a lipidomic “footprint” of molecular changes, which may support reliable diagnostics even at early disease stages, prediction of treatment response and monitoring of treatment success (theranostics). Here, we summarize the latest fluid lipidomics discoveries in SSD-related research, examining the latest strategies to integrate information into multi-systems overviews that generate new perspectives of SSD-related psychosis identification, development, and treatment.

## Introduction

Schizophrenia spectrum disorders (SSD) affect up to one in a hundred individuals and are considered one of the leading causes of disability (1–3). Existing treatment options require a life-long commitment from those affected, vary in their effectiveness, and are accompanied by frequent severe side effects that contribute toward poor patient adherence (4, 5).

Early diagnosis has been shown to lead to better patient outcomes (6). Since several mental disorders, including SSD, emerge during adolescence, considerable effort must be made toward identification and intervention during these formative years. Decades of research into clinical assessment and treatment evaluation have helped develop standardized models for diagnosing various mental conditions (7, 8). However, even these well-defined clinical standards display high comorbidity between and high heterogeneity within their classifications, and are based solely on somehow subjective clinical assessments of complex symptoms, impeding an accurate clinical diagnosis. Additionally, these current syndrome-focused classifications for SSD fit poorly to young people presenting with subthreshold mental health conditions (9, 10). Especially in the early stages, the clinical features of SSD may be non-specific, overlap with those of other mental health conditions (e.g., depression, anxiety, substance abuse, and bipolar disorder), or not yet sufficiently severe to meet traditional diagnostic criteria (10–12).

Precision medicine is a growing approach to tackle the root cause behind any given disease/condition through consideration of an individual's biological proneness and their response to treatment (13). This approach has significant implications for the field of psychiatry as it challenges the traditional application of clinical concepts in mental health with new diagnostic and therapeutic approaches that explore the pathophysiology (14). The task is immense and requires multiple meta-data sets across biological systems to be matched with multimodal information (e.g., age, gender, clinical characteristics, neuropsychological features, or brain imaging data).

Advancements in omics technologies offer new and exciting research perspectives for biosignatures (biomarkers) and molecular insight to delineate the complexity of SSD etiopathologies. Several genome-wide association studies (GWAS) have shown that common variants and expression patterns are associated with an increased risk of SSD, pointing to gene-expression pathways involved in calcium-channel regulation, microRNA-mediated gene regulation, and immune function (15–17). However, variation patterns of gene-expression in these pathways have also been identified between SSD and other mental health conditions, including autism, bipolar, and major depressive disorders (18–23), highlighting the limitation of investigating a singular “omics” branch to unravel biological complexity contributing to altered physiology. Likewise, SSD pathology is not confined to a single data type. It requires an interdisciplinary understanding of perturbations in gene expression, alongside proteomic and metabolic dysregulation, with evidence for the latter describing influences on mechanisms involved in synaptic function, glucose metabolism, and regulatory processes involved in lipid homeostasis (24–28).

The role of lipids in SSD is of particular interest due to their characteristically high concentration in the brain, second only to adipose tissue. In the central nervous system (CNS), lipids play a major role as interfaces for cell signaling, secondary messengers, sources of energy, and neurotrophic support (29–32). Lipidomics, a subset of metabolomics, is the focused characterization and quantification of lipids (33). Several reviews have looked at the contribution of impaired lipid metabolism in the development of neurological disorders, including SSD, and found irregularities for several classes of lipids across various brain regions (28, 33–35). Lipids are also viewed as favorable candidates for neurological biomarkers given their prevalence across various accessible biofluids, including blood [lipids represent up to 70% of all detectable metabolites in serum (36)], saliva, and cerebrospinal fluid (CSF). CSF is the fluid that is anatomically most closely linked to the CNS and is considered a biological source of information that provides a true reflection of the pathological manifestations occurring in the brain (37).

Peripheral lipid analysis is already clinically established to measure cardiovascular disease (CVD) risk from a lipid panel of sterols and triglycerides (TG) (38). Given the comorbidity of CVD and mental health conditions (39–41), these lipid assays have been adapted for linked investigations, showing that elevated plasma TG and very-low-density lipoprotein (VLDL) levels are strongly associated with various cognitive domains in individuals with schizophrenia (SCZ) (42). In addition, high cholesterol levels appear to be positively associated with depression in adults with psychosis (43). Meta-analyses have revealed low levels of high-density lipoproteins (HDL) and hypertriglyceridemia in individuals with multiple episodes of SCZ (44, 45). A few theranostics studies have also examined lipid response to treatment. HDL levels appear to increase along with improvement in negative symptoms following antipsychotic treatment (46), while supplementation with omega-3 polyunsaturated fatty acids (ω-3 PUFA) decreased TG levels in adolescents at ultra-high risk for psychosis (47).

Analytical detection is a main challenge when ascertaining the lipidome's broader function(s) and their potential as biomarkers in SSD. To put the problem into perspective, Lipid Maps^®^, a consortium dedicated to mapping the mammalian lipidome, has compiled (to date) 47,189 unique lipid structures, and this number continues to grow daily (48). Although it is not yet possible to detect and quantify every single lipid in a given sample, broad lipid profiling relies almost exclusively on mass spectrometry to simultaneously identify and measure as many lipids as possible in a given biofluid, which also supports clinical and pathogenic investigations (49). In particular, liquid chromatography coupled with tandem mass spectrometry (LC-MS/MS) has been instrumental toward the identification of several lipid mediators involved in the pathogenesis of SSD (50–53), and as a practical application to explore lipidomic response toward antipsychotics (54, 55). For recent reviews of technological platforms and progress using LC-MS/MS for lipidomics, including studies investigating SSD, please refer to (56–60). Our review focuses on reporting the latest applications employing LC-MS/MS for lipidomics in fluidic biomarker assessment of SSD and potential strategies for integration with other omics datasets to better define conditions, prognosis, and treatment response.

## Lipidomics Investigations into SSD and Psychosis Fluid Biomarkers

Here we review the recent applications of LC-MS/MS, employed either in targeted (e.g., multiple reaction monitoring; MRM) or global analyses to measure lipid expression profiles in SSD and related psychoses in human peripheral fluids over the last 5 years. Our review includes an overview of studies published in English that identified probable fluid lipid biomarkers and their potential role in disease characterization, informing diagnosis, and predicting and monitoring treatment outcomes (theranostics). Studies not involving fluid markers and not utilizing LC-MS/MS were excluded from this review. A literature search of the Google Scholar, PubMed, and Web of Science (ISI Web of Knowledge) electronic databases was conducted due to their readily available access to scholarly literature from vast biomedical databases. Search terms “lipidomics,” “fluid biomarkers,” “schizophrenia,” and “psychosis” were used to filter the selection of studies for this review, dating back to January 1st, 2017 (5 years from 2021). For earlier reviews detailing fluid lipid investigations into SSD, please refer to (28, 34, 61). The reference lists of selected articles were hand-searched to retrieve any additional relevant published papers. Literature searches were performed independently by co-authors TAC and BJ, with accepted studies verified by CR and FML. We concluded on seventeen studies that fitted our search criteria, with one paper excluded post-screening due to the study not providing sufficient detail to evaluate lipidomic response from the variety of medications administered. A summary of lipid changes observed in these studies is presented in Table 1, with classification and nomenclature based on the Lipid Maps^®^ consortium and their structural database (48, 79). Details on individual lipid changes are provided in Supplementary Table S1 (where available).

**Table 1:** Summary of identified lipid alterations in SSD and related psychoses.

**Lipid class (regulation)**	**Condition(s) examined**	**Fluid**	**Factor examined (diagnosis/prognosis/treatment)**	**Integrated study (Y/N)**	**References**
• AA (↓/↑) • DHA (↓) • EPA (↓/↑) • EA (↑) • EDA (↑)	SCZ	Serum	Diagnosis	N	(62)
• AA (↓/↑) • DHA (↓) • EPA (↑) • EA (↓) • EDA (↓) • LA (↓)	SCZ	Serum	Treatment—mixed	N	
AEA (↑)	SCZ	CSF	Diagnosis	N	(63)
AEA (↑)	SCZ patients with binocular depth inversion illusion	Serum	Diagnosis	N	
• AEA (↑) • OEA (↓)	SCZ and schizo-affective disorder	Serum	Diagnosis	N	(64)
AEA (↓/↑)	SCZ and schizo-affective disorder	Serum	Prognosis—association with symptoms	N	
• LPC (↓/↑) • LPE (↓/↑) • PC (↓/↑) • PE (↓) • SM (↑)	SCZ	Serum	Diagnosis	N	(65)
• FFA (↓/↑) • LPC (↓/↑) • LPE (↓/↑) • PC (↓/↑) • PE (↓/↑) • SM (↓/↑)	SCZ	Serum	Diagnosis	N	(66)
• LPC (↑) • PC (↓) • SM (↓)	FEP	Serum	Diagnosis	N	(67)
• LPC (↑) • PC (↑) • SM (↓)	FEP	Serum	Treatment	N	
• FFA (↓) • LPC (↑) • LPE (↑) • PC (↓/↑) • PE (↓/↑)	SCZ	Plasma	Diagnosis	N	(68)
FA (↓/↑)	SCZ	Serum	Diagnosis	N	(69)
FFA (↓)	SCZ	Plasma	Diagnosis	N	(70)
FFA (↓)	SCZ and affective psychosis disorders	Plasma	Diagnosis	N	
FFA (↑)	Affective psychosis disorders	Plasma	Diagnosis	N	
• CE (↑) • LPC (↑) • PC (↑) • SM (↑) • TG (↓)	Psychotic disorders	Plasma	Prognosis	N	(71)
• TG (↑) • SM (↑) • PI (↑) • PC (↑) • LPC (↑) • Cer (↑) • CE (↑)	CHR-P	Serum	Prognosis	N	(72)
TG (↑)	FEP and CHR-P	Plasma	Prognosis—association with BMI	N	(73)
• AEA (↑) • PEA (↑)	SCZ	Plasma	Diagnosis	N	(74)
• 2-AG (↓) • AEA (↑) • LEA (↑) • OEA (↑) • PEA (↑) • PC (↓/↑)	FEP	Serum	Diagnosis	N	(75)
• 2-AG (↑) • AEA (↓) • LEA (↓) • OEA (↓) • PEA (↓) • PC (↓/↑)	FEP	Serum	Treatment	N	
• Cer (↓/PR) • DG (↓/PR) • PA (↓/PR) • PC (↓/PR) • PG (↓/PR) • PS (↓/PR) • SM (↓/PR) • TG (↓/PR) • PC (↓/↑/GR) • PE (↑/GR) • PI-Cer (↓/GR)	SCZ	Plasma	Treatment—Risperidone	N	(76)
• PS (↓/PR) • PA (↓/PR) • PS (↓/PR) • PA (↑/GR) • PC (↑/GR) • PG (↑/GR)	SCZ	Plasma	Treatment—Olanzapine	N	
• PS (↓/PR) • Cer (↓/PR) • PG (↑/GR) • PI (↑/GR)	SCZ	Plasma	Treatment—Quetiapine	N	
• CE (↑) • LPC (↓/↑) • PC (↓) • PE (↓) • SM (↑) • TG (↑)	SCZ	Plasma	Diagnosis	N	(77)
• CE (↓) • Cer (↓) • FA (↓) • LPC (↓) • PC (↓) • SM (↓) • TG (↓)	SCZ	Plasma	Treatment—mixed	N	
• PC (↑) • LPC (↑)	Psychosis (pre-clinical)	Plasma	Diagnosis/prognosis	Y	(78)

*AA, Arachidonic acid; AEA, Anandamide; CE, Cholesterol ester; Cer, Ceramide; Ch, Cholesterol; DG, Diglyceride; DHA, Docosahexaenoic acid; EA, Ethanolamide; EDA, Eicosadienoic acid; EPA, Eicosapentaenoic acid; LA, Linoleic acid; FA, Fatty acid; FFA, Free fatty acid; GR, Good responder; LPC, lysophosphatidylcholine; LPE, lysophosphatidylethanolamine; PA, Phosphatidic acid; PC, Phosphatidylcholine; PE, Phosphatidylethanolamine; PEA, Palmitoylethanolamide; PG, Phosphatidylglycerol; PI, Phosphatidylinositol; PI-Cer, Ceramide phosphoinositol; PR, Poor responder; PS, Phosphatidylserine; SM, Sphingomyelin; TG, Triglyceride*.

### Fluid Lipidomics in the Characterization of SSD

**Figure 1:**
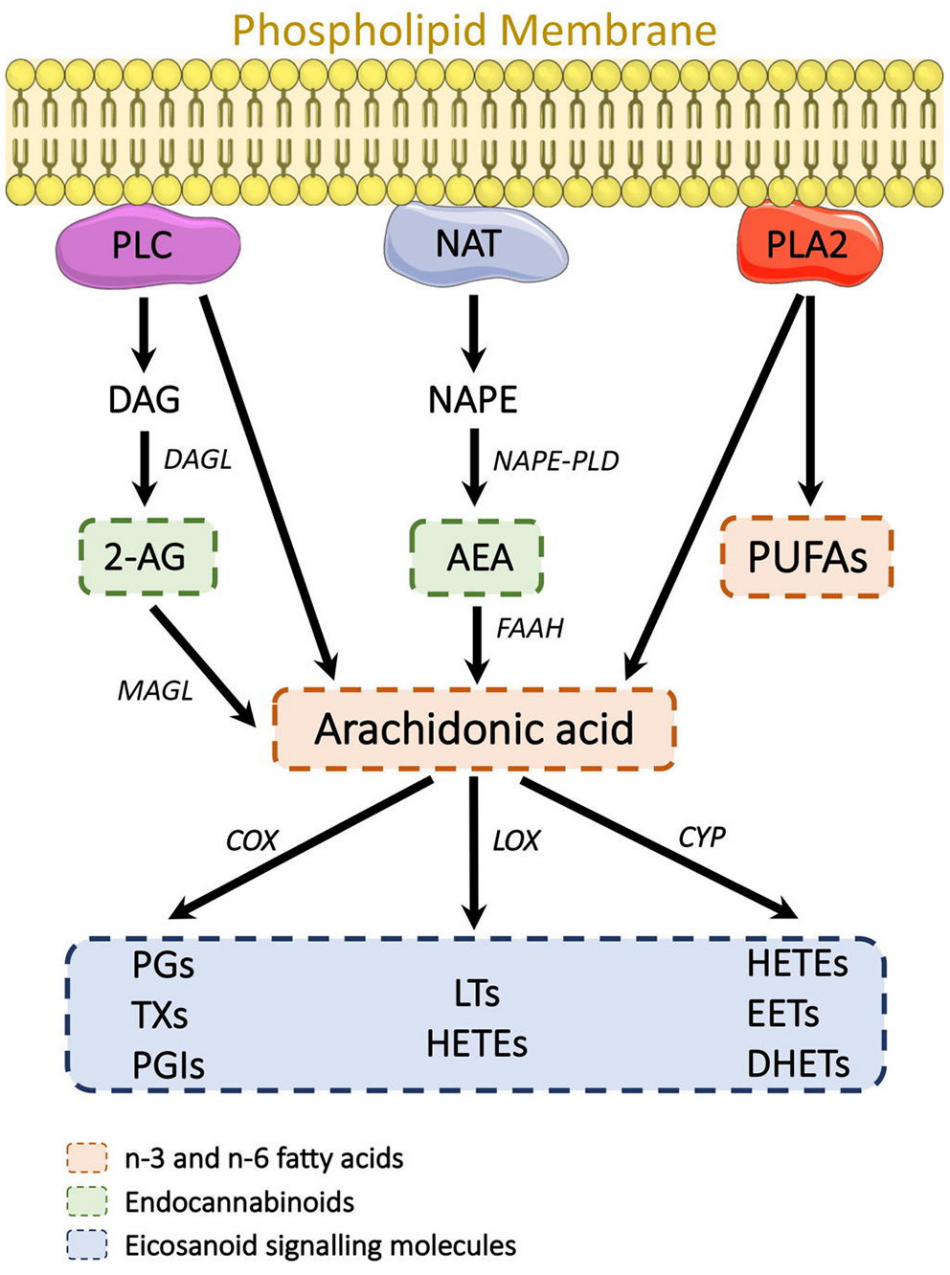
Known lipid-derived mechanisms associated with SCZ etiology. The eCBs, FFAs, and eicosanoids all originate from the breakdown of membrane phospholipids. 2-AG is derived from PLC cleavage of PIP_2_ into DAG and subsequent hydrolysis into 2-AG. NAT mediates phosphatidylethanolamine and phosphatidylcholine breakdown into NAPE, which is then converted by NAPE-PLD into AEA. PLA_2_ mediates the conversion of phospholipids into FFA. AA is generated from membrane phospholipid hydrolysis by PLC and PLA_2_. AA is then further broken down in eicosanoids (PG, TX, PGI, LT, HETE, EET, and DHET) by COX, LOX, and CYP enzymes. 2-AG, 2-Arachidonoylglycerol; AA, Arachidonic acid; AEA, Anandamide; COX, cyclooxygenase; CYP, cytochrome; DAG, Diacylglycerol; DHET, dihydroxyeicosatrienoic acid; EET, eicosatrienoic acid; FFA, Free fatty acid; HETE, Monohydroxyeicosatetraenoic acids; LOX, Lipoxygenase; LT, Leukotriene; NAT, N-acetyl transferase; NAPE, N-arachidonyl phosphatidylethanolamine; NAPE-PLD, N-acyl phosphatidylethanolamine-specific phospholipase D; PG, Prostaglandin; PGI, Prostacyclin; PLA_2_, Phospholipase A2; PLC, Phospholipase C; PIP_2_, Phosphatidylinositol 4,5-biphosphate; TX, Thromboxane.

A systematic study into potential SCZ biomarkers in serum by Wang *et al*. (62) observed twenty-three analytes significantly altered in individuals with SCZ at baseline levels compared to healthy controls, belonging to bioactive lipid mediator eicosanoids, derived from the release of arachidonic acid (AA), an essential PUFA which is commonly released by membrane phospholipids (80) (Figure 1). Significant increases were also observed in anandamide (AEA) and the related N-acylethanolamine (NAE): oleoylethanolamine (OEA). AEA is a lipid-derived agonist of the cannabinoid receptors and, as one of the two endocannabinoids (eCBs), a major component of the endocannabinoid system (ECS), a neuromodulatory system found throughout the CNS and periphery. eCBs are well-known to be perturbed during the pathogenesis of SCZ, in particular AEA, found elevated in the CSF of young adults with first-episode SCZ (51, 52), and more recently by Reuter *et al*. (63), along with evidence of AEA in serum serving as peripheral marker of its neuronal activity. Furthermore, Potvin *et al*. (64) found increased plasma AEA and OEA levels in individuals with SCZ presenting to psychiatric emergency settings, i.e., individuals experiencing an acute psychotic episode, with AEA and OEA levels decreasing again by the time of discharge. Equally, AEA and OEA, along with AA, formed the panel of biomarkers in Wang and colleagues' study that best differentiated SCZ individuals from their control counterparts (62).

Further studies in which Wang *et al*. (65) employed untargeted LC-MS/MS profiling identified several membrane phospholipids, and their lyso-derivatives, as potential biomarkers in SCZ, forming a secondary panel of lipids that may discriminate individuals with SCZ from healthy controls. Phospholipids are among the most abundant lipid species in the CNS and critical constituents of biological membranes. They play an integral role in maintaining membrane structure and function, particularly in the integrity of myelin, which is pivotal to electrical impulse propagation, where perturbations can directly impact neurotransmission (81, 82). Multiple lines of evidence implicate that the etiology of SCZ is associated with increased breakdown of membrane phospholipids to free fatty acids (FFAs), including arachidonic acid and lysophospholipids, primarily through the hydrolysis actions of phospholipase A_2_ (PLA2) (83, 84), which formed the biochemical basis for the membrane phospholipid concept in SCZ (85) (Figure 1).

Further, Wang *et al*. (66) undertook a phospholipid targeted approach, optimizing LC-MS/MS detection for over 150 phospholipids and FFAs. Their findings emphasized expression tendencies in phospholipids were associated with their degree of saturation and chain length, with similar results observed by Leppik *et al*. (67) investigating drug-naïve first-episode psychosis (FEP) individuals. In this study, declining phospholipid levels were in connection with observed increases in long-chain acylcarnitine (ACs) intermediates (e.g., AC 18:2), and these complex lipid profile abnormalities may be interlinked to form part of the underlying pathology (67).

In addition, Wang *et al*. (66) observed that polyunsaturated lysophosphatidylcholines (LPC) were elevated, indicating either a weakened LPC catalysis by lecithin-cholesterol acyltransferases (LCAT) or increased PLA2 breakdown. MRM analysis by Liu *et al*. (68) partly validated these dysregulated phospholipid homeostasis findings, identifying several FFA, AC intermediates, along with phosphatidylcholine (PC) and LPC in plasma of individuals with SCZ compared to matched controls, adding to their biomarker candidacy.

Perturbations to observed FFAs originate, in part, from membrane phospholipid metabolism, potentially making them a source of biomarkers that could describe the intricate glycerophospholipids lipid distortions in SCZ. Yang *et al*. (69) explored the potential of FFAs as peripheral markers of membrane dysfunctions in SCZ, along with lipid markers previously identified in their metabolic study, and found elevated levels of monounsaturated fatty acids (oleic acid, palmitoleic acid) and ω-3 PUFA (docosatetraenoic acid, AA, and linoleic acid). The authors hypothesized that these changes occur as a consequence of enhanced lipolysis and β-oxidation.

To explore the possibility that FFAs perturbations are specific to SSD, Zhou *et al*. (70) investigated FFA levels in individuals with first-episode SCZ or affective psychosis (bipolar disorder or major depression with psychotic features) and healthy controls. Zhou *et al*. identified several imbalances in FFA levels in SCZ, predominantly a decline in saturated and ω-3 fatty acids. They speculated that the declining plasma levels were due to increased oxidative stress early in SCZ development, leading to membrane phospholipid deficits and, in turn, increased fatty acid demand for cell membrane regeneration. Importantly, changes in FFA were specific to SCZ. Thus, they can be considered as potential SSD-specific biomarkers. Furthermore, the reduction in n-6 fatty acids was almost explicitly observed in a subgroup of SCZ patients with blunted niacin-induced skin flushing symptoms, demonstrating the potential capacity of lipidomics to differentiate specific conditions within the spectrum of SSD.

### Lipids as Pre-clinical Indicators of Psychosis

Though prodromal clinical features of psychosis have long been described and investigated (86), deciphering the actual individual risk for the transition from clinically high risk (CHR) to first-episode still remains a clinical challenge (87), with less than one-third of individuals progressing within 3 years (88). Longitudinal studies in lipidomics have observed lipid aberrations at pre-clinical stages that may give potential to their application as early indicators.

The prospective Avon Longitudinal Study of Parents and Children (ALSPAC) collected biological samples and health data from children and followed them up until age 18 (89). O'Gorman *et al*. (71) used ALSPAC plasma samples from individuals clinically diagnosed with a psychotic disorder at 18 years of age, corresponding plasma samples collected 7 years prior to diagnosis, as well as plasma from age- and BMI-matched controls, and employed a metabolomic (including lipidomic) assessment. At age 11, children with a later diagnosis of psychosis already had significantly elevated levels of a subset of lipids (32, with eight lipids remaining significant after correction for multiple comparisons), including various membrane phospholipids [LPCs, PCs, and sphingomyelin (SM)], whose altered metabolism has been suggested as a pathogenic mechanism for SCZ (90). At age 18, a subset of lipids was decreased in those diagnosed with a psychotic disorder compared to controls; however, none reached significance after correction for multiple comparisons (71). In addition, their follow-up study, integrating lipidomic with proteomic data, revealed interconnections between these omics systems and is discussed in more detail below.

A 5-year follow-up study into global lipid profiles of individuals with a CHR state for psychosis and healthy controls revealed several TGs elevated in CHR subjects at baseline measurements, i.e., independent of later transition (72). This is consistent with findings of a recent study, analyzing the association between weight gain in FEP and CHR individuals to predict the development of cardiometabolic comorbidities, showing a positive association between weight gain in these individuals and the levels of TGs with low carbon number and double-bond count (73). Interestingly, TGs with low carbon and double-bond count are commonly associated with non-alcoholic fatty liver disease and type II diabetes, suggesting that weight gain is more likely to occur in FEP individuals with high liver fat content rather than dietary lipid content. In addition, Dickens *et al*. utilized machine learning algorithms to distill sub-groups of CHR individuals to better predict their future clinical outcome and successfully identified individuals who subsequently developed psychosis based on reduced levels of ether phospholipids (72).

Evidence also points toward the ECS as a regulator of CHR individuals developing psychosis, with Koethe *et al*. (74) reporting peripheral levels of eCBs AEA and 2-arachidonylglycerol (2-AG) in monozygotic twins discordant for SCZ were directly related to their risk for developing psychosis. They observed that twins with lower levels of AEA and 2-AG in plasma were more prone to develop a psychotic disorder during their 5-year follow-up assessment period, along with their suggestive use for examination in a prognostic fashion (74).

Most recently, Parksepp *et al*. (75) conveyed a direct and dynamic influence of membrane phospholipids on ECS regulation in SSD. In their 5-year investigation, levels of peripheral ECS regulators and NAEs were examined along with their phospholipid precursors. Elevated serum levels of NAEs, including AEA, were exhibited in antipsychotic-naïve patients along with a significant increase in their respective phospholipid precursors, which resolved post-antipsychotic treatment, including a reduction of NAE synthesis. 2-AG, which is derived from a separate lipid precursor pathway, was reduced in antipsychotic-naïve patients, subsequently displaying a significant increase at the 5-year evaluation time-point, which also included a rise of lipids responsible for its formation.

### Lipids in Theranostics

There is increasing optimism that the progressive construction of omics systems will refine mechanisms for selecting personalized treatment and monitoring intervention-derived shifts in response to physiological reaction(s). Lipid involvement in neurological functions and evidence for their disturbance in the prodromal stages of psychiatric disorders infers that pharmacological intervention will likely, in turn, elicit a lipid response.

While an approach to monitor treatment effects of quetiapine on eCBs and related FAEs did not reveal a link (91), Aquino *et al*. (92) conducted a broader lipidomics study to determine the effects of antipsychotic treatment in acutely ill SCZ individuals. The aim was to predict how patients would respond to specific treatments. Their retrospective study identified multiple lipid compounds in individuals treated with olanzapine, risperidone, and quetiapine that differed between responders and those with little to no improvement. However, molecular pathways for these analytes have yet to be verified. Subsequent evaluation of lipid response before and after treatment identified risperidone as the antipsychotic with the largest impact on lipid expression (76). Specifically, de Almeida *et al*. (76) found that the lipid class phosphatidylserine was uniquely associated with a poor response to all treatments, while changes in other lipids associated with treatment response were antipsychotic-specific. Of all lipid classes, glycerophosphocholines (PCs) were most affected by antipsychotics. This finding is consistent with another study showing that 7 months after baseline assessments in antipsychotic-naïve FEP individuals, certain PCs were up-regulated by antipsychotics (67).

Yan *et al*. (77) also aimed to identify lipid alterations indicating the onset of SCZ and subsequent response to treatment. Various lipids were significantly dysregulated in antipsychotic-naïve individuals with SCZ compared to healthy controls, including several new SM, AC, and ceramide species. Eight weeks of antipsychotic treatment (various drugs, not further specified) affected lipid levels in multiple ways. Some lipid levels did not change; some were restored by treatment and reached levels comparable to healthy controls, while other lipid levels began declining or decreased even further in their abundance.

The side effects of typical and atypical antipsychotics are a recurring problem, resulting in poor adherence to treatment plans. Therefore, research into new therapeutics with fewer adverse effects is of immense importance. Cannabidiol (CBD) is being considered as a viable candidate for a next-generation antipsychotic, owing to its promising efficacy in SCZ and moderate side effects (93–96). Although underlying mechanisms remain still conjectural, growing evidence suggests CBD may exert its antipsychotic properties, at least in part, *via* lipid signaling in the ECS (93–96). A systematic examination of lipids in response to CBD might shed light on their physiological relevance to CBD efficacy, particularly given recent dynamics investigating the consequence of phospholipidomic shifts on altered ECS function (75). To the best of our knowledge, so far, no clinical investigations have explored peripheral lipid profiles in response to CBD, either as a stand-alone or adjunct therapy with antipsychotics.

### Integration Omics Provides New Insight Into Complex Interrelationships in SSD and Psychosis

Biomarkers that are unique, stable, and reliable are considered an important step toward personalized medicine. However, a major obstacle in their development has been poor reproducibility and unclear interpretation of their biological mechanism. The growing body of evidence, including that already mentioned, has shown that multiple omics systems are involved in SSD and that a holistic approach is needed to capture their involvement in disease etiology more accurately.

Previous studies pointed to dysregulation of both the lipidome and proteome early in adolescence, even before the onset of psychotic symptoms (71, 97). A follow-up study used plasma samples from 12-year old children of the ALSPAC cohort who were reported to develop a psychotic disorder at 18 years of age (78), examining the interrelationship between these systems within the same individuals before the onset of psychotic experiences. Multivariate network analysis showed high connectivity between several phosphatidylcholines with the complement pathway protein vitronectin and members of the coagulation cascade (plasminogen and heparin cofactor 2), supporting previous literature on their involvement in SCZ (85, 98, 99). Notably, this study represents one of the first to link lipidomics to other omics datasets and highlights that metabolic disturbances (including an interaction between lipidome and proteome) may contribute to early vulnerability in the development of psychotic disorders.

In a recent study, Campeau *et al*. (100) performed a multi-omics analysis of plasma samples from individuals with SCZ and healthy controls (aged 28–74 years) to examine age- and disease-related effects on various proteins and metabolites. The majority of altered metabolites between SCZ and healthy controls were classified as lipids. Their network analysis revealed that these metabolic alterations were highly interconnected with several proteomic influences to apolipoproteins (Apo), a lipid transporter family responsible for regulating the transport and metabolism of lipids both in the CNS and periphery, whose disruption has also been observed in serum of patients with SCZ and metabolic syndrome (101). In addition, through advanced machine learning approaches, their study identified multiple age-related risk factors for SCZ, with several linked to well-known CVD biomarkers, which are themselves risk factors and major contributors to premature death in SCZ (42–45).

Finally, at the beginning of this year, Wang *et al*. (102) constructed a pathway-based, integrated network generated from metabolomics and transcriptomics data from human plasma to investigate their functional interrelationship in the pathogenesis of SCZ. Their endogenous interactive network analysis uncovered important differences in phospholipid, amino acid and energy metabolism, and observed latent pathway interactions between metabolites belonging to these pathways and genes with associated functions, including HMGCLL1 (ketogenesis), ALDH4A (proline/hydroxyproline metabolism), and PLA2G4D (phospholipase A2) which may play an essential role in SCZ. Diagnostic formulas for SCZ were established based on metabolomic and transcriptomic results, and their predictive ability was scrupulously confirmed. The results showed that the formulas could be used to distinguish different states of disease and health with good accuracy, indicating the relevance of the identified variations in the metabolome and transcriptome.

## Discussion

SSD are heterogeneous psychiatric syndromes whose large variability in origin, modes of mechanism, and clinical expression impede accurate diagnosis and treatment. Lipids are essential for the maintenance of neuronal function, and it is widely accepted that in SSD, lipid metabolism is disturbed. Therefore, they are promising biomarker candidates, especially in light of the fact that the field of lipidomics is developing exponentially after advancements in mass spectrometry have enabled the generation of global lipid profiles in high-throughput scenarios that can accommodate differing clinical and pathological conditions (e.g., antipsychotic-naïve, first episode, therapeutic response).

In this review, we focused on lipidomic studies in biofluid tissue, which could be utilized as indicative markers of neurological deficits in SSD. A vast majority of the studies reported fluid biomarker candidates associated with phospholipid metabolism and PUFAs, both critical regulators of cerebral membrane function and fluidity (103), which have also been reported as irregularities across various brain regions in people with SCZ, reviewed in (28, 33–35). Responses toward molecular SSD targets of interest derived from these phospholipid precursors (Figure 1) were also observed. These included regulators of the ECS—a promising pathway for therapeutic intervention (104), as well as AA and AA-derived eicosanoids whose effects on inflammatory response are well-established in SSD, with evidence to suggest that decreasing these inflammatory biomarkers may improve clinical outcome (105).

It is promising that the lipid classes showing dysregulation were comparable between the CNS and peripheral tissue, particularly given their biological importance in the CNS, as well as the brain's reliance on the transport of PUFAs from circulatory systems (e.g., blood) for subsequent incorporation into cerebral phospholipid membranes (30). The similarities may reflect biological pathways or processes conserved between these systems, strengthening the use of these fluid lipid changes as surrogate biomarkers of neuropathology in SSD.

In addition, the findings described suggest that impaired lipid homeostasis, including subsequent effects on lipid-derived signaling mediators generated from these precursors, precedes the onset of psychosis, with the changes observed over the course of pathogenesis being dynamic and able to be reversed following treatment. These outlooks support future investigations into the restoration of lipid signaling as an intervention strategy, as well as lipidomic evaluations on current and future (e.g., CBD) drug effects. If successful, specific lipid biomarkers could be used to predict and monitor treatment response.

SSD is also associated with higher rates of mortality, linked to its co-morbidities such as diabetes, obesity, metabolic syndrome, hyperlipidemia, and CVD (54, 106), all of which are characterized by changes in lipid metabolism. TGs were also identified as clinically high-risk factors for SSD and associated with FEP (72, 73). These dietary lipids have been well-described as markers of increased mortality resulting from the co-morbidities that develop during SSD. Now, it may be that an increased frequency of these lipids in co-morbid conditions reflects a greater risk of developing SSD itself.

Despite mounting evidence of lipids involvement in SSD, the power to extract clinically applicable lipid biomarkers remains a challenge. Many probable lipid candidates are not exclusive to SSD. Similar perturbations to phospholipids and TGs identified in this review have also been reported in various neurological disorders and co-morbidities, reviewed in Alves *et al*. (107). This may reflect shared underlying pathological processes in the various neurological and metabolic disorders.

Alternatively, results may be limited to the lipidomic analysis employed. Unbiased lipidomic profiling with LC-MS/MS is conventionally measured using data-dependent acquisition, a lipid screening format that biases abundant lipids. Targeted screening methods, such as MRM, aid the identification of lipids of interest, down to picomolar concentrations, and were employed to identify eCBs and eicosanoid disruptions in SSD etiology (51, 52, 62, 69, 74, 75). Data-independent acquisition is an alternative global lipidomic approach, providing a more diverse lipidomics profile. This was employed by Yan *et al*. (77) and led to the discovery of new sphingolipid fluid markers, the third largest component of cell membranes behind phospholipids and cholesterol, and critically important regulators of cell signaling, development, and maintenance.

Consensus and design of studies are also plausible limitations in recent clinical lipidomics investigations. Recruitment and drop-outs are an ongoing challenge for research studies, made even more difficult when participants with various conditions are needed. This may have limitations on the size of available cohorts, categorization of sub-groups, as well as the biofluid available for analysis. This latter point may explain why we have identified only a single CSF investigation into SSD over the past 5 years, and that perceived invasiveness of lumbar punctures may hinder its broader use (108). Reliance on blood, arguably the next closest anatomical fluid to the brain after CSF, is also problematic, as the global lipid profiles were reported to be considerably different between the two biofluids (109).

Though some of these limitations cannot currently be overcome, alternatives are being explored that incorporate omics systems collectively to help uncover interrelated pathways that lead to new disease associations not previously identified with a single modality. This integrative approach may also interpret new connections between tissue types, including relationships between CSF and blood lipid levels, not seen by chance or simple associations, leading to the discovery of new biomarkers and therapeutics. Although the integration of lipidomics datasets is still in its infancy, some of the analyses reported here have applied integrative strategies and demonstrated new functional relationships of lipids with proteins, metabolic pathways, and gene expression, previously unknown in psychiatric conditions (78, 100, 102).

Omics datasets, with or without integration, also present unique analytical challenges regarding data interpretation. This task is particularly challenging in SSD, as omics associations could be interconnected with a multitude of clinical phenotypes. However, new data analysis strategies involving artificial intelligence and machine learning algorithms are now being employed to accommodate these multiple variables. For example, Madrid-Gambin *et al*. (78) utilized computational algorithms to identify patterns representative of different metabolic phenotypes in their study cohort, while Dickens *et al*. (72) used machine learning to identify new early-onset risk factors. Integrating omics data with machine learning strategies not only delineates the complexity of SSD but also advances precision medicine in psychiatry.

Since Horrobin's original insight into lipids and SSD (85), our knowledge has expanded exponentially, with advances in lipidomics technologies, such as LC-MS/MS, driving a more in-depth understanding of lipids involvement in SSD. While elucidating the role of lipid disruptions in SSD pathophysiology remains an ongoing investigation, bioinformatics tools are becoming increasingly available to pursue more integrative approaches that are beginning to unravel these complex processes through a true systems biology approach.

## Author Contributions

TAC and BJ completed the background literature search and wrote the first draft of the article. All authors listed have made a substantial, direct, and intellectual contribution to the work and approved it for publication.

## Funding

This work was supported by DVCR start up grant to FML from the University of Sydney.

## Conflict of Interest

FML is a shareholder of curantis UG (Ltd.). CR is a shareholder of lero bioscience UG (Ltd). The remaining authors declare that the research was conducted in the absence of any commercial or financial relationships that could be construed as a potential conflict of interest.

## Publisher's Note

All claims expressed in this article are solely those of the authors and do not necessarily represent those of their affiliated organizations, or those of the publisher, the editors and the reviewers. Any product that may be evaluated in this article, or claim that may be made by its manufacturer, is not guaranteed or endorsed by the publisher.

## References

[1] Vos T Abajobir AA Abate KH Abbafati C Abbas KM Abd-Allah F. Global, regional, and national incidence, prevalence, and years lived with disability for 328 diseases and injuries for 195 countries, 1990–2016: a systematic analysis for the Global Burden of Disease Study 2016. Lancet. (2017) 390:1211–59. 10.1016/S0140-6736(17)32154-2 28919117 PMC5605509

[2] World Health Organisation. Schizophrenia World Health Organisation. (2019). Available from: https://www.who.int/news-room/fact-sheets/detail/schizophrenia (accessed November 15, 2021).

[3] Rossler W Salize HJ van Os J Riecher-Rossler A. Size of burden of schizophrenia and psychotic disorders. Eur Neuropsychopharmacol. (2005) 15:399–409. 10.1016/j.euroneuro.2005.04.009 15925493

[4] Nose M Barbui C Tansella M. How often do patients with psychosis fail to adhere to treatment programmes? A systematic review. Psychol Med. (2003) 33:1149–60. 10.1017/S0033291703008328 14580069

[5] Miyamoto S Duncan G Marx C Lieberman J. Treatments for schizophrenia: a critical review of pharmacology and mechanisms of action of antipsychotic drugs. Mol Psychiatry. (2005) 10:79–104. 10.1038/sj.mp.4001556 15289815

[6] Larsen TK Melle I Auestad B Haahr U Joa I Johannessen JO. Early detection of psychosis: positive effects on 5-year outcome. Psychol Med. (2011) 41:1461–9. 10.1017/S0033291710002023 20942996

[7] World Health Organisation. The ICD-10 Classification of Mental and Behavioural Disorders: Clinical Descriptions and Diagnostic Guidelines. Geneva: World Health Organisation (1993).

[8] American Psychological Association. Diagnostic and Statistical Manual of Mental Disorders. 5th ed. Washington, DC: American Psychiatric Publishing (2013).

[9] Shah JL Scott J McGorry PD Cross SPM Keshavan MS Nelson B. Transdiagnostic clinical staging in youth mental health: a first international consensus statement. World Psychiatry. (2020) 19:233–42. 10.1002/wps.20745 32394576 PMC7215079

[10] Hickie IB Scott EM Cross SP Iorfino F Davenport TA Guastella AJ. Right care, first time: a highly personalised and measurement-based care model to manage youth mental health. Med J Aust. (2019) 211 Suppl 9:S3–S46. 10.5694/mja2.50383 31679171

[11] Buckley PF Miller BJ Lehrer DS Castle DJ. Psychiatric comorbidities and schizophrenia. Schizophr Bull. (2009) 35:383–402. 10.1093/schbul/sbn135 19011234 PMC2659306

[12] Keshavan MS Morris DW Sweeney JA Pearlson G Thaker G Seidman LJ. A dimensional approach to the psychosis spectrum between bipolar disorder and schizophrenia: the Schizo-Bipolar Scale. Schizophr Res. (2011) 133:250–4. 10.1016/j.schres.2011.09.005 21996268 PMC3381911

[13] Ashley EA. The precision medicine initiative: a new national effort. JAMA. (2015) 313:2119–20. 10.1001/jama.2015.3595 25928209

[14] Fernandes BS Williams LM Steiner J Leboyer M Carvalho AF Berk M. The new field of 'precision psychiatry'. BMC Med. (2017) 15:80. 10.1186/s12916-017-0849-x 28403846 PMC5390384

[15] Schizophrenia Working Group of the Psychiatric Genomics C. Biological insights from 108 schizophrenia-associated genetic loci. Nature. (2014) 511:421–7. 10.1038/nature13595 25056061 PMC4112379

[16] Collier DA Eastwood BJ Malki K Mokrab Y. Advances in the genetics of schizophrenia: toward a network and pathway view for drug discovery. Ann NY Acad Sci. (2016) 1366:61–75. 10.1111/nyas.13066 27111133

[17] Giusti-Rodriguez P Sullivan PF. The genomics of schizophrenia: update and implications. J Clin Invest. (2013) 123:4557–63. 10.1172/JCI66031 24177465 PMC3809776

[18] Smeland OB Bahrami S Frei O Shadrin A O'Connell K Savage J. Correction: Genome-wide analysis reveals extensive genetic overlap between schizophrenia, bipolar disorder, and intelligence. Mol Psychiatry. (2020) 25:914. 10.1038/s41380-019-0456-7 31308466

[19] Witt SH Streit F Jungkunz M Frank J Awasthi S Reinbold CS. Genome-wide association study of borderline personality disorder reveals genetic overlap with bipolar disorder, major depression and schizophrenia. Transl Psychiatry. (2017) 7:e1155. 10.1038/tp.2017.115 28632202 PMC5537640

[20] Kushima I Aleksic B Nakatochi M Shimamura T Okada T Uno Y. Comparative analyses of copy-number variation in autism spectrum disorder and schizophrenia reveal etiological overlap and biological insights. Cell Rep. (2018) 24:2838–56. 10.1016/j.celrep.2018.08.022 30208311

[21] Musliner KL Mortensen PB McGrath JJ Suppli NP Hougaard DM Bybjerg-Grauholm J. Association of polygenic liabilities for major depression, bipolar disorder, and schizophrenia with risk for depression in the danish population. JAMA Psychiatry. (2019) 76:516–25. 10.1001/jamapsychiatry.2018.4166 30698613 PMC6495355

[22] O'Connell KS McGregor NW Lochner C Emsley R Warnich L. The genetic architecture of schizophrenia, bipolar disorder, obsessive-compulsive disorder and autism spectrum disorder. Mol Cell Neurosci. (2018) 88:300–7. 10.1016/j.mcn.2018.02.010 29505902

[23] Pain O Dudbridge F Cardno AG Freeman D Lu Y Lundstrom S. Genome-wide analysis of adolescent psychotic-like experiences shows genetic overlap with psychiatric disorders. Am J Med Genet B Neuropsychiatr Genet. (2018) 177:416–25. 10.1002/ajmg.b.32630 29603866 PMC6001485

[24] Holmes E Tsang TM Huang JT Leweke FM Koethe D Gerth CW. Metabolic profiling of CSF: evidence that early intervention may impact on disease progression and outcome in schizophrenia. PLoS Med. (2006) 3:e327. 10.1371/journal.pmed.0030327 16933966 PMC1551919

[25] Nascimento JM. Martins-de-Souza D. The proteome of schizophrenia. NPJ Schizophr. (2015) 1:14003. 10.1038/npjschz.2014.3 27336025 PMC4849438

[26] Oresic M Seppanen-Laakso T Sun D Tang J Therman S Viehman R. Phospholipids and insulin resistance in psychosis: a lipidomics study of twin pairs discordant for schizophrenia. Genome Med. (2012) 4:1. 10.1186/gm300 22257447 PMC3334549

[27] Pickard BS. Schizophrenia biomarkers: translating the descriptive into the diagnostic. J Psychopharmacol. (2015) 29:138–43. 10.1177/0269881114566631 25601396

[28] Schneider M Levant B Reichel M Gulbins E Kornhuber J Muller CP. Lipids in psychiatric disorders and preventive medicine. Neurosci Biobehav Rev. (2017) 76(Pt B):336–62. 10.1016/j.neubiorev.2016.06.002 27317860

[29] Bruce KD Zsombok A Eckel RH. Lipid processing in the brain: a key regulator of systemic metabolism. Front Endocrinol. (2017) 8:60. 10.3389/fendo.2017.00060 28421037 PMC5378716

[30] Hamilton JA Hillard CJ Spector AA Watkins PA. Brain uptake and utilization of fatty acids, lipids and lipoproteins: application to neurological disorders. J Mol Neurosci. (2007) 33:2–11. 10.1007/s12031-007-0060-1 17901539

[31] Cermenati G Mitro N Audano M Melcangi RC Crestani M De Fabiani E. Lipids in the nervous system: from biochemistry and molecular biology to patho-physiology. Biochim Biophys Acta. (2015) 1851:51–60. 10.1016/j.bbalip.2014.08.011 25150974

[32] Piomelli D Astarita G Rapaka R. A neuroscientist's guide to lipidomics. Nat Rev Neurosci. (2007) 8:743–54. 10.1038/nrn2233 17882252

[33] Wenk MR. The emerging field of lipidomics. Nat Rev Drug Discov. (2005) 4:594–610. 10.1038/nrd1776 16052242

[34] Sethi S Hayashi MA Barbosa BS Pontes JG Tasic L Brietzke E. Lipidomics, biomarkers, and schizophrenia: a current perspective. Metabolomics. 965:265–90. 10.1007/978-3-319-47656-8_11 28132184

[35] Zhao YY Cheng XL Lin RC. Lipidomics applications for discovering biomarkers of diseases in clinical chemistry. Int Rev Cell Mol Biol. (2014) 313:1–26. 10.1016/B978-0-12-800177-6.00001-3 25376488

[36] Psychogios N Hau DD Peng J Guo AC Mandal R Bouatra S. The human serum metabolome. PLoS ONE. (2011) 6:e16957. 10.1371/journal.pone.0016957 21359215 PMC3040193

[37] Ghosh A Nishtala K. Biofluid lipidome: a source for potential diagnostic biomarkers. Clin Transl Med. (2017) 6:22. 10.1186/s40169-017-0152-7 28639235 PMC5479868

[38] Cardiovascular Disease: Risk Assessment and Reduction Including Lipid Modification. London: National Institute for Health and Care Excellence: Guidelines (2016).

[39] Tickell AM Rohleder C Ho N McHugh C Jones G Song YJC. Identifying pathways to early-onset metabolic dysfunction, insulin resistance and inflammation in young adult inpatients with emerging affective and major mood disorders. Early Interv Psychiatry. (2021). 10.1111/eip.13260. [Epub ahead of print]. 34852406

[40] Hagenaars SP Coleman JRI Choi SW Gaspar H Adams MJ Howard DM. Genetic comorbidity between major depression and cardio-metabolic traits, stratified by age at onset of major depression. Am J Med Genet B Neuropsychiatr Genet. (2020) 183:309–30. 10.1016/j.euroneuro.2018.08.008 32681593 PMC7991693

[41] Correll CU Solmi M Veronese N Bortolato B Rosson S Santonastaso P. Prevalence, incidence and mortality from cardiovascular disease in patients with pooled and specific severe mental illness: a large-scale meta-analysis of 3,211,768 patients and 113,383,368 controls. World Psychiatry. (2017) 16:163–80. 10.1002/wps.20420 28498599 PMC5428179

[42] Nandeesha H Keshri N Rajappa M Menon V. Association of hyperglycaemia and hyperlipidaemia with cognitive dysfunction in schizophrenia spectrum disorder. Arch Physiol Biochem. (2020) 1–8. 10.1080/13813455.2020.1839500 33142080

[43] Gohar SM Dieset I Steen NE Mørch RH Iversen TS Steen VM. Association between serum lipid levels, osteoprotegerin and depressive symptomatology in psychotic disorders. Eur Arch Psychiatry Clin Neurosci. (2019) 269:795–802. 10.1007/s00406-018-0897-z 29721726 PMC6739273

[44] Vancampfort D Wampers M Mitchell AJ Correll CU De Herdt A Probst M. A meta-analysis of cardio-metabolic abnormalities in drug naive, first-episode and multi-episode patients with schizophrenia versus general population controls. World Psychiatry. (2013) 12:240–50. 10.1002/wps.20069 24096790 PMC3799255

[45] Mitchell AJ Vancampfort D Sweers K van Winkel R Yu W De Hert M. Prevalence of metabolic syndrome and metabolic abnormalities in schizophrenia and related disorders–a systematic review and meta-analysis. Schizophr Bull. (2013) 39:306–18. 10.1093/schbul/sbr148 22207632 PMC3576174

[46] Gjerde PB Dieset I Simonsen C Hoseth EZ Iversen T Lagerberg TV. Increase in serum HDL level is associated with less negative symptoms after one year of antipsychotic treatment in first-episode psychosis. Schizophr Res. (2018) 197:253–60. 10.1016/j.schres.2017.10.042 29129510

[47] Mossaheb N Papageorgiou K Schäfer MR Becker J Schloegelhofer M Amminger GP. Changes in triglyceride levels in ultra-high risk for psychosis individuals treated with omega-3 fatty acids. Early Interv Psychiatry. (2018) 12:30–6. 10.1111/eip.12275 26362578

[48] Sud M Fahy E Cotter D Brown A Dennis EA Glass CK. LMSD: LIPID MAPS structure database. Nucleic Acids Res. (2007) 35:D527–32. 10.1093/nar/gkl838 17098933 PMC1669719

[49] Ackermann BL Hale JE Duffin KL. The role of mass spectrometry in biomarker discovery and measurement. Curr Drug Metab. (2006) 7:525–39. 10.2174/138920006777697918 16787160

[50] Muguruza C Lehtonen M Aaltonen N Morentin B Meana JJ Callado LF. Quantification of endocannabinoids in postmortem brain of schizophrenic subjects. Schizophr Res. (2013) 148:145–50. 10.1016/j.schres.2013.06.013 23800614

[51] Leweke FM Giuffrida A Wurster U Emrich HM Piomelli D. Elevated endogenous cannabinoids in schizophrenia. Neuroreport. (1999) 10:1665–9. 10.1097/00001756-199906030-00008 10501554

[52] Giuffrida A Leweke FM Gerth CW Schreiber D Koethe D Faulhaber J. Cerebrospinal anandamide levels are elevated in acute schizophrenia and are inversely correlated with psychotic symptoms. Neuropsychopharmacol. (2004) 29:2108–14. 10.1038/sj.npp.1300558 15354183

[53] Schwarz E Prabakaran S Whitfield P Major H Leweke FM Koethe D. High throughput lipidomic profiling of schizophrenia and bipolar disorder brain tissue reveals alterations of free fatty acids, phosphatidylcholines, and ceramides. J Proteome Res. (2008) 7:4266–77. 10.1021/pr800188y 18778095

[54] McEvoy J Baillie RA Zhu H Buckley P Keshavan MS Nasrallah HA. Lipidomics reveals early metabolic changes in subjects with schizophrenia: effects of atypical antipsychotics. PLoS ONE. (2013) 8:e68717. 10.1371/journal.pone.0068717 23894336 PMC3722141

[55] Kaddurah-Daouk R McEvoy J Baillie RA Lee D Yao JK Doraiswamy PM. Metabolomic mapping of atypical antipsychotic effects in schizophrenia. Mol Psychiatry. (2007) 12:934–45. 10.1038/sj.mp.4002000 17440431

[56] Kofeler HC Fauland A Rechberger GN Trotzmuller M. Mass spectrometry based lipidomics: an overview of technological platforms. Metabolites. (2012) 2:19–38. 10.3390/metabo2010019 24957366 PMC3901195

[57] Sethi S Hayashi MA Sussulini A Tasic L Brietzke E. Analytical approaches for lipidomics and its potential applications in neuropsychiatric disorders. World J Biol Psychiatry. (2017) 18:506–20. 10.3109/15622975.2015.1117656 26555297

[58] Wu Z Bagarolo GI Thoroe-Boveleth S Jankowski J. “Lipidomics”: Mass spectrometric and chemometric analyses of lipids. Adv Drug Deliv Rev. (2020) 159:294–307. 10.1016/j.addr.2020.06.009 32553782

[59] Wu Z Shon JC Liu KH. Mass spectrometry-based lipidomics and its application to biomedical research. J Lifestyle Med. (2014) 4:17–33. 10.15280/jlm.2014.4.1.17 26064851 PMC4390758

[60] Wood PL. Mass spectrometry strategies for clinical metabolomics and lipidomics in psychiatry, neurology, and neuro-oncology. Neuropsychopharmacol. (2014) 39:24–33. 10.1038/npp.2013.167 23842599 PMC3857645

[61] Davison J O'Gorman A Brennan L Cotter DR A. systematic review of metabolite biomarkers of schizophrenia. Schizophr Res. (2018) 195:32–50. 10.1016/j.schres.2017.09.021 28947341

[62] Wang D Sun X Yan J Ren B Cao B Lu Q. Alterations of eicosanoids and related mediators in patients with schizophrenia. J Psychiatr Res. (2018) 102:168–78. 10.1016/j.jpsychires.2018.04.002 29674269

[63] Reuter AR Bumb JM Mueller JK Rohleder C Pahlisch F Hanke F. Association of anandamide with altered binocular depth inversion illusion in schizophrenia. World J Biol Psychiatry. (2017) 18:483–8. 10.1080/15622975.2016.1246750 27734750

[64] Potvin S Mahrouche L Assaf R Chicoine M Giguere CE Furtos A. Peripheral endogenous cannabinoid levels are increased in schizophrenia patients evaluated in a psychiatric emergency setting. Front Psychiatry. (2020) 11:628. 10.3389/fpsyt.2020.00628 32695035 PMC7338686

[65] Wang D Cheng SL Fei Q Gu H Raftery D Cao B. Metabolic profiling identifies phospholipids as potential serum biomarkers for schizophrenia. Psychiatry Res. (2019) 272:18–29. 10.1016/j.psychres.2018.12.008 30579177

[66] Wang D Sun X Maziade M Mao W Zhang C Wang J. Characterising phospholipids and free fatty acids in patients with schizophrenia: a case-control study. World J Biol Psychiatry. (2021) 22:161–74. 10.1080/15622975.2020.1769188 32677491

[67] Leppik L Parksepp M Janno S Koido K Haring L Vasar E. Profiling of lipidomics before and after antipsychotic treatment in first-episode psychosis. Eur Arch Psychiatry Clin Neurosci. (2020) 270:59–70. 10.1007/s00406-018-0971-6 30604052

[68] Liu Y Song X Liu X Pu J Gui S Xu S. Alteration of lipids and amino acids in plasma distinguish schizophrenia patients from controls: a targeted metabolomics study. Psychiatry Clin Neurosci. (2021) 75:138–44. 10.1111/pcn.13194 33421228

[69] Yang X Sun L Zhao A Hu X Qing Y Jiang J. Serum fatty acid patterns in patients with schizophrenia: a targeted metabonomics study. Transl Psychiatry. (2017) 7:e1176. 10.1038/tp.2017.152 28742081 PMC5538128

[70] Zhou X Long T Haas GL Cai H Yao JK. Reduced levels and disrupted biosynthesis pathways of plasma free fatty acids in first-episode antipsychotic-naive schizophrenia patients. Front Neurosci. (2020) 14:784. 10.3389/fnins.2020.00784 32848558 PMC7403507

[71] O'Gorman A Suvitaival T Ahonen L Cannon M Zammit S Lewis G. Identification of a plasma signature of psychotic disorder in children and adolescents from the Avon longitudinal study of parents and children (ALSPAC) cohort. Transl Psychiatry. (2017) 7:e1240. 10.1038/tp.2017.211 28949339 PMC5639252

[72] Dickens AM Sen P Kempton MJ Barrantes-Vidal N Iyegbe C Nordentoft M. Dysregulated lipid metabolism precedes onset of psychosis. Biol Psychiatry. (2021) 89:288–97. 10.1016/j.biopsych.2020.07.012 32928501

[73] Lamichhane S Dickens AM Sen P Laurikainen H Borgan F Suvisaari J. Association between circulating lipids and future weight gain in individuals with an at-risk mental state and in first-episode psychosis. Schizophr Bull. (2021) 47:160–9. 10.1093/schbul/sbaa087 32609372 PMC7825089

[74] Koethe D Pahlisch F Hellmich M Rohleder C Mueller JK Meyer-Lindenberg A. Familial abnormalities of endocannabinoid signaling in schizophrenia. World J Biol Psychiatry. (2019) 20:117–25. 10.1080/15622975.2018.1449966 29521179

[75] Parksepp M Haring L Kilk K Koch K Uppin K Kangro R. The expanded endocannabinoid system contributes to metabolic and body mass shifts in first-episode schizophrenia: a 5-year follow-up study. Biomedicines. (2022) 10:243. 10.3390/biomedicines10020243 35203453 PMC8869544

[76] de Almeida V Alexandrino GL Aquino A Gomes AF Murgu M Dobrowolny H. Changes in the blood plasma lipidome associated with effective or poor response to atypical antipsychotic treatments in schizophrenia patients. Prog Neuro Psychopharmacol Biol Psychiatry. (2020) 101:109945. 10.1016/j.pnpbp.2020.109945 32304808

[77] Yan L Zhou J Wang D Si D Liu Y Zhong L. Unbiased lipidomic profiling reveals metabolomic changes during the onset and antipsychotics treatment of schizophrenia disease. Metabolomics. (2018) 14:80. 10.1007/s11306-018-1375-3 30830385

[78] Madrid-Gambin F Föcking M Sabherwal S Heurich M English JA O'Gorman A. Integrated lipidomics and proteomics point to early blood-based changes in childhood preceding later development of psychotic experiences: evidence from the Avon longitudinal study of parents and children. Biol Psychiatry. (2019) 86:25–34. 10.1016/j.biopsych.2019.01.018 30878195 PMC6579334

[79] Fahy E Subramaniam S Murphy RC Nishijima M Raetz CR Shimizu T. Update of the LIPID MAPS comprehensive classification system for lipids. J Lipid Res. (2009) 50(Suppl.):S9–14. 10.1194/jlr.R800095-JLR200 19098281 PMC2674711

[80] Rocha PN Plumb TJ Coffman TM. Eicosanoids: lipid mediators of inflammation in transplantation. Springer Semin Immunopathol. (2003) 25:215–27. 10.1007/s00281-003-0132-4 12955468

[81] Fernandis AZ Wenk MR. Membrane lipids as signaling molecules. Curr Opin Lipidol. (2007) 18:121–8. 10.1097/MOL.0b013e328082e4d5 17353659

[82] du Bois TM Deng C Huang XF. Membrane phospholipid composition, alterations in neurotransmitter systems and schizophrenia. Prog Neuropsychopharmacol Biol Psychiatry. (2005) 29:878–88. 10.1016/j.pnpbp.2005.04.034 16005134

[83] Gattaz WF Hubner CV Nevalainen TJ Thuren T Kinnunen PK. Increased serum phospholipase A2 activity in schizophrenia: a replication study. Biol Psychiatry. (1990) 28:495–501. 2223919

[84] Pettegrew JW Keshavan MS Panchalingam K Strychor S Kaplan DB Tretta MG. Alterations in brain high-energy phosphate and membrane phospholipid metabolism in first-episode, drug-naive schizophrenics. A pilot study of the dorsal prefrontal cortex by *in vivo* phosphorus 31 nuclear magnetic resonance spectroscopy. Arch Gen Psychiatry. (1991) 48:563–8. 10.1001/archpsyc.1991.01810300075011 1898445

[85] Horrobin DF. The membrane phospholipid hypothesis as a biochemical basis for the neurodevelopmental concept of schizophrenia. Schizophr Res. (1998) 30:193–208. 10.1016/S0920-9964(97)00151-5 9589514

[86] Schultze-Lutter F Debbane M Theodoridou A Wood SJ Raballo A Michel C. Revisiting the basic symptom concept: toward translating risk symptoms for psychosis into neurobiological targets. Front Psychiatry. (2016) 7:9. 10.3389/fpsyt.2016.00009 26858660 PMC4729935

[87] Koutsouleris N Dwyer DB Degenhardt F Maj C Urquijo-Castro MF Sanfelici R. Multimodal machine learning workflows for prediction of psychosis in patients with clinical high-risk syndromes and recent-onset depression. JAMA Psychiatry. (2021)78:195–209. 10.1001/jamapsychiatry.2020.3604 33263726 PMC7711566

[88] Fusar-Poli P Bonoldi I Yung AR Borgwardt S Kempton MJ Valmaggia L. Predicting psychosis: meta-analysis of transition outcomes in individuals at high clinical risk. Arch Gen Psychiatry. (2012) 69:220–9. 10.1001/archgenpsychiatry.2011.1472 22393215

[89] Boyd A Golding J Macleod J Lawlor DA Fraser A Henderson J. Cohort profile: the 'children of the 90s'–the index offspring of the Avon longitudinal study of parents and children. Int J Epidemiol. (2013) 42:111–27. 10.1093/ije/dys064 22507743 PMC3600618

[90] Horrobin DF Glen AI Vaddadi K. The membrane hypothesis of schizophrenia. Schizophr Res. (1994) 13:195–207. 10.1016/0920-9964(94)90043-4 7841132

[91] Potvin S Kouassi E Lipp O Bouchard RH Roy MA Demers MF. Endogenous cannabinoids in patients with schizophrenia and substance use disorder during quetiapine therapy. J Psychopharmacol. (2008) 22:262–9. 10.1177/0269881107083816 18308802

[92] Aquino A Alexandrino GL Guest PC Augusto F Gomes AF Murgu M. Blood-based lipidomics approach to evaluate biomarkers associated with response to olanzapine, risperidone, and quetiapine treatment in schizophrenia patients. Front Psychiatry. (2018) 9:209. 10.3389/fpsyt.2018.00209 29887809 PMC5982405

[93] Leweke F Piomelli D Pahlisch F Muhl D Gerth C Hoyer C. Cannabidiol enhances anandamide signaling and alleviates psychotic symptoms of schizophrenia. Transl Psychiatry. (2012) 2:e94. 10.1038/tp.2012.15 22832859 PMC3316151

[94] Leweke FM Mueller JK Lange B Fritze S Topor CE Koethe D. Role of the endocannabinoid system in the pathophysiology of schizophrenia: implications for pharmacological intervention. CNS Drugs. (2018) 32:605–19. 10.1007/s40263-018-0539-z 30022465

[95] Leweke FM Rohleder C Gerth CW Hellmich M Pukrop R Koethe D. Cannabidiol and amisulpride improve cognition in acute schizophrenia in an explorative, double-blind, active-controlled, randomized clinical trial. Front Pharmacol. (2021) 12:614811. 10.3389/fphar.2021.614811 33995015 PMC8117353

[96] Rohleder C Muller JK Lange B Leweke FM. Cannabidiol as a potential new type of an antipsychotic. A critical review of the evidence. Front Pharmacol. (2016) 7:422. 10.3389/fphar.2016.00422 27877130 PMC5099166

[97] English JA Lopez LM O'Gorman A Focking M Hryniewiecka M Scaife C. Blood-based protein changes in childhood are associated with increased risk for later psychotic disorder: evidence from a nested case-control study of the ALSPAC longitudinal birth cohort. Schizophr Bull. (2018) 44:297–306. 10.1093/schbul/sbx075 29036721 PMC5814944

[98] Hoirisch-Clapauch S Amaral OB Mezzasalma MA Panizzutti R Nardi AE. Dysfunction in the coagulation system and schizophrenia. Transl Psychiatry. (2016) 6:e704. 10.1038/tp.2015.204 26731441 PMC5068878

[99] Sekar A Bialas AR de Rivera H Davis A Hammond TR Kamitaki N. Schizophrenia risk from complex variation of complement component 4. Nature. (2016) 530:177–83. 10.1038/nature16549 26814963 PMC4752392

[100] Campeau A Mills RH Stevens T Rossitto LA Meehan M Dorrestein P. Multi-omics of human plasma reveals molecular features of dysregulated inflammation and accelerated aging in schizophrenia. Mol Psychiatry. (2022) 27:1217–25. 10.1038/s41380-021-01339-z 34741130 PMC9054664

[101] Boiko AS Mednova IA Kornetova EG Semke AV Bokhan NA Loonen AJM. Apolipoprotein serum levels related to metabolic syndrome in patients with schizophrenia. Heliyon. (2019) 5:e02033. 10.1016/j.heliyon.2019.e02033 31317083 PMC6611937

[102] Wang T Li P Meng X Zhang J Liu Q Jia C. An integrated pathological research for precise diagnosis of schizophrenia combining LC-MS/(1)H NMR metabolomics and transcriptomics. Clin Chim Acta. (2022) 524:84–95. 10.1016/j.cca.2021.11.028 34863699

[103] Assies J Mocking RJ Lok A Ruhe HG Pouwer F Schene AH. Effects of oxidative stress on fatty acid- and one-carbon-metabolism in psychiatric and cardiovascular disease comorbidity. Acta Psychiatr Scand. (2014) 130:163–80. 10.1111/acps.12265 24649967 PMC4171779

[104] Leweke FM Mueller JK Lange B Rohleder C. Therapeutic potential of cannabinoids in psychosis. Biol Psychiatry. (2016) 79:604–12. 10.1016/j.biopsych.2015.11.018 26852073

[105] Keller WR Kum LM Wehring HJ Koola MM Buchanan RW Kelly DL. review of anti-inflammatory agents for symptoms of schizophrenia. J Psychopharmacol. (2013) 27:337–42. 10.1177/0269881112467089 23151612 PMC3641824

[106] Lambert TJ Velakoulis D Pantelis C. Medical comorbidity in schizophrenia. Med J Aust. (2003) 178:S67–70. 10.5694/j.1326-5377.2003.tb05311.x 12720526

[107] Alves MA Lamichhane S Dickens A McGlinchey A Ribeiro HC Sen P. Systems biology approaches to study lipidomes in health and disease. Biochim Biophys Acta Mol Cell Biol Lipids. (2021) 1866:158857. 10.1016/j.bbalip.2020.158857 33278596

[108] Day GS Rappai T Sathyan S Morris JC. Deciphering the factors that influence participation in studies requiring serial lumbar punctures. Alzheimers Dement. (2020) 12:e12003. 10.1002/dad2.12003 32211499 PMC7085282

[109] Saito K Hattori K Hidese S Sasayama D Miyakawa T Matsumura R. Profiling of cerebrospinal fluid lipids and their relationship with plasma lipids in healthy humans. Metabolites. (2021) 11. 10.3390/metabo11050268 PMC8146161 33923144

