# Supplementary material for: Current State of Fluid Lipid Biomarkers for Personalized Diagnostics and Therapeutics in Schizophrenia Spectrum Disorders and Related Psychoses: A Narrative Review
Source: Front Psychiatry. 2022 May 27;13:885904. doi: 10.3389/fpsyt.2022.885904 (PMC9197191; doi:10.3389/fpsyt.2022.885904)
Supplement: Supplementary file 1 [file Data_Sheet_1.pdf]

Supplementary table S1: Individual lipid alterations in SSD and related psychoses.

Lipid name	Change in levels	Involvement	Patient details	Biofluid	REF
AA	Downregulated	SCZ patients vs. healthy controls	Drug naïve/drug free (4 wks off) SCZ patients	Serum	Wang, et al. (64)
PGE2	Downregulated				
PGA2	Downregulated				
PGF2α	Downregulated				
PGJ2	Downregulated				
TXB2	Downregulated				
11-dehydro-TXB2	Downregulated				
11,12-DHET	Upregulated				
14,15-DHET	Upregulated				
20-carboxy-AA	Upregulated				
LTB4	Downregulated				
12-HpETE	Downregulated				
12-HETE	Downregulated				
15-HETE	Upregulated				
8-HETE	Upregulated				
11-HETE	Downregulated				
DHA	Downregulated				
13-HDoHE	Downregulated				
EPA	Downregulated				
12-HEPE	Upregulated				
AEA	Upregulated				
OEA	Upregulated				
15-KEDE	Upregulated				
PGE2	Upregulated	Pre- vs. post- antipsychotics	Antipsychotics not specified		
PGF2α	Upregulated				
TXB2	Upregulated				
11-dehydro-TXB2	Upregulated				
11,12-DHET	Downregulated				
14,15-DHET	Downregulated				
20-carboxy-AA	Downregulated				
5-KETE	Downregulated				
12-HETE	Upregulated				
4-HDoHE	Downregulated				
7-HDoHE	Downregulated				
12-HEPE	Upregulated				
AEA	Downregulated				
OEA	Downregulated				
15-KEDE	Downregulated				
9-HpODE	Downregulated				
9-HODE	Downregulated				
9-KODE	Downregulated				
13-HpODE	Downregulated				
13-HODE	Downregulated				
13-KODE	Downregulated				
9,10-DiHOME	Downregulated				
AEA	Upregulated	SCZ patients vs. healthy controls	FEP, antipsychotic naïve	CSF	Reuter, et al. (66)
AEA	Upregulated	SCZ with binocular depth illusion inversion vs. healthy controls	FEP, antipsychotic naïve	Serum	
AEA	Upregulated	SSD patients vs. healthy controls	Patients on various treatments	Serum	Potvin, et al. (67)
OEA	Upregulated				
AEA	Positively correlated with depressive symptoms	SSD patient symptoms	Patients on various treatments	Serum	
AEA	Negatively correlated with sleep efficiency				
LPC(0:0/18:0)d	Downregulated				
LPC(14:0)d	Downregulated				
LPC(15:0)	Downregulated				
LPC(17:0)d	Downregulated				
LPC(17:1)	Downregulated				
LPC(18:0)d	Downregulated				
LPC(19:0)d	Downregulated				
LPC(20:0)d	Downregulated				
LPC(20:1)	Downregulated				
LPC(22:6)	Upregulated				
LPE(16:0)d	Upregulated				
LPE(20:0)	Downregulated				
LPE(20:4)d	Upregulated				
PC(15:0/18:1)	Upregulated				
PC(16:0/16:0)d	Upregulated				

Supplementary table S1: Individual lipid alterations in SSD and related psychoses.

Lipid name	Change in levels	Involvement	Patient details	Biofluid	REF
PC(16:0/18:1)d	Upregulated	SCZ patients vs. healthy controls	Mix of FEP and drug-free recurrent patients	Serum	Wang, et al. (68)
PC(16:0/22:5)	Upregulated				
PC(16:1/16:0)d	Upregulated				
PC(18:2/17:0)d	Downregulated				
PC(18:2/17:1)	Downregulated				
PC(18:2/18:2)d	Downregulated				
PC(20:3/16:0)d	Downregulated				
PC(20:4/14:0)	Downregulated				
PC(20:4/18:0)d	Upregulated				
PC(20:4/18:2)d	Downregulated				
PC(20:4/P-18:1)	Downregulated				
PC(22:6/14:0)d	Downregulated				
PC(O-16:0/18:2)d	Downregulated				
PC(O-16:0/20:3)	Downregulated				
PC(O-16:0/20:4)d	Downregulated				
PC(O-18:0/20:4)	Downregulated				
PC(O-18:0/22:6)	Downregulated				
PC(P-16:0/18:2)d	Downregulated				
PC(P-18:1/18:1)	Downregulated				
PE(O-16:0/20:4)	Downregulated				
PE(O-16:0/22:5)	Downregulated				
PE(O-16:0/22:6)	Downregulated				
PE(O-18:0/20:4)	Downregulated				
PE(P-16:0/18:2)	Downregulated				
PE(P-16:0/20:4)d	Downregulated				
PE(P-16:0/22:6)	Downregulated				
PE(P-18:0/18:2)d	Downregulated				
PE(P-18:0/20:4)d	Downregulated				
PE(P-18:0/22:6)	Downregulated				
PE(P-18:1/22:6)	Downregulated				
SM(d18:0/18:1)	Upregulated				
SM(d18:0/18:2)	Upregulated				
SM(d18:1/16:0)d	Upregulated				
SM(d18:1/24:1)d	Upregulated				
SM(d18:2/22:1)	Upregulated				
SM(d18:2/24:1)	Upregulated				
FFA(14:0)	Upregulated				
FFA(16:0)	Upregulated				
FFA(16:1)	Upregulated				
FFA(17:0)	Upregulated				
FFA(17:1)	Upregulated				
FFA(18:0)	Upregulated				
FFA(18:1)	Upregulated				
FFA(20:0)	Upregulated				
FFA(20:1)	Upregulated				
FFA(20:2)	Upregulated				
FFA(20:3)	Upregulated				
FFA(20:4)	Downregulated				
FFA(22:1)	Upregulated				
FFA(22:2)	Upregulated				
FFA(22:4)	Upregulated				
FFA(24:1)	Upregulated				
LPC(0:0/14:0)	Downregulated				
LPC(0:0/16:0)	Downregulated				
LPC(0:0/18:0)	Downregulated				
LPC(0:0/18:3)	Downregulated				
LPC(0:0/19:0)	Downregulated				
LPC(0:0/20:0)	Downregulated				
LPC(0:0/20:2)	Downregulated				
LPC(0:0/20:4)	Upregulated				
LPC(0:0/22:6)	Upregulated				
LPC(14:0)	Downregulated				
LPC(16:0)	Downregulated				
LPC(17:0)	Downregulated				
LPC(17:1)	Downregulated				
LPC(18:0)	Downregulated				
LPC(18:1)	Downregulated				
LPC(18:3)	Downregulated				
LPC(19:0)	Downregulated				
LPC(20:0)	Downregulated				
LPC(20:2)	Downregulated				
LPC(20:4)	Upregulated				
LPC(22:0)	Downregulated				
LPC(22:6)	Upregulated				

Supplementary table S1: Individual lipid alterations in SSD and related psychoses.

Lipid name	Change in levels	Involvement	Patient details	Biofluid	REF
LPC(24:0)	Downregulated	SCZ patients vs. healthy controls	Mix of FEP and drug-free recurrent patients	Serum	Wang, et al. (74)
LPE(0:0/16:0)	Upregulated				
LPE(0:0/18:0)	Downregulated				
LPE(0:0/20:4)	Upregulated				
LPE(0:0/22:6)	Upregulated				
LPE(16:0)	Upregulated				
LPE(18:0)	Downregulated				
LPE(18:1)	Downregulated				
LPE(20:4)	Upregulated				
LPE(22:6)	Upregulated				
PC(14:0/18:2)	Downregulated				
PC(16:0/14:0)	Upregulated				
PC(16:0/16:0)	Upregulated				
PC(16:0/18:1)	Upregulated				
PC(16:0/20:4)	Upregulated				
PC(16:1/14:0)	Upregulated				
PC(16:1/16:0)	Upregulated				
PC(18:0/16:0)	Downregulated				
PC(18:0/18:1)	Downregulated				
PC(18:0/18:2)	Downregulated				
PC(18:0/20:3)	Downregulated				
PC(18:2/16:0)	Upregulated				
PC(18:2/17:0)	Downregulated				
PC(18:2/18:2)	Downregulated				
PC(18:2/19:0)	Downregulated				
PC(20:0/18:1)	Downregulated				
PC(20:3/16:0)	Downregulated				
PC(20:4/14:0)	Downregulated				
PC(20:4/18:0)	Upregulated				
PC(20:4/18:2)	Downregulated				
PC(20:4/20:4)	Downregulated				
PC(22:5/16:0)	Upregulated				
PC(22:6/14:0)	Downregulated				
PC(22:6/16:0)	Upregulated				
PC(22:6/18:0)	Upregulated				
PC(O-16:0/16:1)	Upregulated				
PC(O-16:0/18:2)	Downregulated				
PC(O-16:0/20:4)	Downregulated				
PC(O-16:0/22:6)	Downregulated				
PC(O-18:0/16:0)	Downregulated				
PC(O-18:0/20:4)	Downregulated				
PC(P-16:0/18:2)	Downregulated				
PC(P-18:0/18:1)	Downregulated				
PC(P-18:0/18:2)	Downregulated				
PC(P-18:0/20:4)	Downregulated				
PC(P-18:0/22:6)	Downregulated				
PE(16:0/18:1)	Upregulated				
PE(18:0/18:1)	Upregulated				
PE(18:2/18:0)	Downregulated				
PE(18:2/18:1)	Downregulated				
PE(20:4/18:0)	Upregulated				
PE(22:6/16:0)	Upregulated				
PE(22:6/16:0)	Upregulated				
PE(22:6/P-16:0)	Downregulated				
PE(22:6/P-18:0)	Downregulated				
PE(P-16:0/20:4)	Downregulated				
PE(P-16:0/22:5)	Downregulated				
PE(P-18:0/18:2)	Downregulated				
PE(P-18:0/20:4)	Downregulated				
SM(d16:1/24:1)	Downregulated				
SM(d17:1/24:1)	Upregulated				
SM(d18:0/16:0)	Downregulated				
SM(d18:1/17:0)	Downregulated				
SM(d18:1/18:0)	Upregulated				
SM(d18:1/18:1)	Upregulated				
SM(d18:1/19:0)	Upregulated				
SM(d18:1/20:0)	Downregulated				
SM(d18:1/22:0)	Downregulated				
SM(d18:1/24:0)	Downregulated				
SM(d18:1/24:1)	Upregulated				
SM(d18:2/24:1)	Upregulated				
SM(d19:1/20:0)	Downregulated				
LysoPC-a-C20:4	Upregulated				
PC-aa-C30:0	Downregulated				
PC-aa-C32:1	Downregulated				
PC-aa-C32:2	Downregulated				
PC-aa-C34:2	Downregulated				
PC-aa-C34:3	Downregulated				
PC-aa-C34:4	Downregulated				

Supplementary table S1: Individual lipid alterations in SSD and related psychoses.

Lipid name	Change in levels	Involvement	Patient details	Biofluid	REF
PC-aa-C36:1	Downregulated	FEP patients vs. healthy controls	FEP patients	Serum	Leppik, et al. (75)
PC-aa-C36:2	Downregulated				
PC-aa-C36:3	Downregulated				
PC-aa-C36:6	Downregulated				
PC-aa-C38:3	Downregulated				
PC-ae-C34:2	Downregulated				
PC-ae-C36:2	Downregulated				
PC-ae-C36:3	Downregulated				
PC-ae-C40:2	Downregulated				
PC-ae-C40:4	Downregulated				
SM-C20:2	Downregulated				
LysoPC-a-C14:0	Upregulated	Before vs. after antipsychotics	FEP patients (administered various antipsychotics)	Serum	
LysoPC-a-C20:3	Upregulated				
PC-aa-C32:2	Upregulated				
PC-aa-C34:3	Upregulated				
PC-aa-C34:4	Upregulated				
PC-aa-C36:1	Upregulated				
PC-aa-C36:2	Upregulated				
PC-aa-C36:3	Upregulated				
PC-aa-C36:6	Upregulated				
PC-aa-C38:3	Upregulated				
PC-aa-C40:5	Upregulated				
SM-(OH)-C16:1	Downregulated				
SM-C18:0	Downregulated				
FFA 16:2	Downregulated	SCZ patients vs. healthy controls	Mix of drug-naïve and treated patients	Plasma	Liu, et al. (76)
FFA 18:2	Downregulated				
FFA 18:3	Downregulated				
FFA 20:4	Downregulated				
FFA 22:6	Downregulated				
LPC 16:0	Upregulated				
LPE 16:1 SN1	Upregulated				
LPE 16:1 SN2	Upregulated				
LPE 22:5	Upregulated				
PC(O-34:2)	Downregulated				
PC 32:1	Upregulated				
PE(O-34:3)	Downregulated				
PE(O-36:6)	Downregulated				
PE 34:2	Upregulated				
BCFA C14.0.iso	Downregulated	SCZ patients vs. healthy controls	Drug naïve/drug free (4 wks off) SCZ patients	Serum	Yang, et al. (77)
BCFA C16.0.iso	Downregulated				
BCFA C18.0.iso	Upregulated				
MFA C12.1.*cis*.11	Upregulated				
MFA C14.1.*cis*.9	Upregulated				
MFA C16.1.*cis*.9	Upregulated				
MFA C18.1.*cis*.9	Upregulated				
MFA C20.1.*cis*.11	Upregulated				
MFA C22.1.*cis*.13	Upregulated				
MFA C24.1.*cis*.15	Upregulated				
OCFA C15.0	Downregulated				
OCFA C15.0.iso	Downregulated				
OCFA C17.0	Upregulated				
OCFA C17.0.iso	Upregulated				
OCFA C17.1.*cis*.10	Upregulated				
OCFA C19.0	Upregulated				
OCFA C19.1.*cis*.10	Upregulated				
OCFA	Upregulated				
OCFA C21.0	Downregulated				
OCFA C21.1.*cis*.12	Upregulated				
OCFA C23.0	Downregulated				
OCFA C23.1.*cis*.14	Upregulated				
SFA C12.0	Downregulated				
SFA C14.0	Upregulated				
SFA C16.0	Upregulated				
SFA C18.0	Upregulated				
SFA C20.0	Upregulated				
SFA C22.0	Downregulated				
SFA C24.0	Downregulated				
SFA C8.0	Upregulated				
TFA C14.1.trans.9	Upregulated				
TFA C16.1.trans.9	Upregulated				
TFA C18.2.trans.9.12	Upregulated				
ω-3 PUFA	Upregulated				
ω-3 PUFA	Downregulated				
ω-3 PUFA	Upregulated				
ω-3 PUFA	Upregulated				
ω-6 PUFA	Upregulated				
ω-6 PUFA	Upregulated				
ω-6 PUFA	Upregulated				
ω-6 PUFA	Upregulated				

Supplementary table S1: Individual lipid alterations in SSD and related psychoses.

Lipid name	Change in levels	Involvement	Patient details	Biofluid	REF
ω-6 PUFA	Upregulated				
ω-6 PUFA	Upregulated				
ω-6 PUFA	Upregulated				
FA(16:0)	Downregulated	First episode SCZ patients vs healthy controls	Drug naïve/drug free patients	Plasma	Zhou, et al. (78)
FA(18:1n9c)	Downregulated				
FA(20:2n6)	Downregulated				
FA(20:4n6)	Downregulated				
FA(22:6n3)	Downregulated				
FA(16:0)	Downregulated in SCZ	First episode SCZ patients vs affective psychosis (bipolar/depression)	Drug naïve/drug free patients	Plasma	
FA(18:0)	Downregulated in SCZ				
FA(22:0)	Downregulated in SCZ				
FA(16:1n7c)	Downregulated in SCZ				
FA(17:1)	Downregulated in SCZ				
FA(18:1n7)	Downregulated in SCZ				
FA(20:4n6)	Downregulated in SCZ				
FA(22:5n3)	Downregulated in SCZ				
CE(18:2)	Upregulated	Patients with psychotic disorder vs. healthy controls	Children at 12 years with psychotic disorders at 18 years	Plasma	O'Gorman, et al. (83)
CE(18:2)+unknown	Upregulated				
LPC(16:1)	Upregulated				
LPC(18:1)	Upregulated				
LPC(18:2)	Upregulated				
LPC(20:3)	Upregulated				
LPC(20:4)	Upregulated				
PC(16:0e/18:1(9Z))	Upregulated				
PC(30:0)	Upregulated				
PC(32:0)	Upregulated				
PC(32:1)	Upregulated				
PC(32:2)	Upregulated				
PC(34:2)	Upregulated				
PC(36:2)	Upregulated				
PC(36:3)	Upregulated				
PC(36:4	Upregulated				
PC(38:2)	Upregulated				
PC(38:4)	Upregulated				
PC(40:6)	Upregulated				
PC(O-32:0)	Upregulated				
PC(O-32:1)	Upregulated				
PC(O-34:2)	Upregulated				
PC(O-34:3)	Upregulated				
PC(O-36:2)	Upregulated				
PC(O-36:3)	Upregulated				
PC(O-38:6)	Upregulated				
SM(d18:0/16:0)	Upregulated				
SM(d18:1/24:0)	Upregulated				
SM(d41:1)	Upregulated				
SM(t34:1)+H	Upregulated				
TG(56:7)	Downregulated				
TG(56:2)	Upregulated	CHR patients vs. healthy controls	Mix of drug-naïve and treated patients	Serum	Dickens, et al. (85)
TG(55:5)	Upregulated				
TG(54:7)	Upregulated				
TG(54:2)	Upregulated				
TG(54:1)	Upregulated				
TG(53:2)	Upregulated				
TG(51:4)	Upregulated				
TG(51:3)	Upregulated				
TG(51:2)	Upregulated				
TG(51:1)	Upregulated				
TG(50:3)	Upregulated				
TG(50:1)	Upregulated				
TG(49:3)	Upregulated				
TG(49:2)	Upregulated				
TG(49:1)	Upregulated				
TG(48:3)	Upregulated				
TG(18:2/22:5/16:0)	Upregulated				
TG(18:2/18:2/18:2) or	Upregulated				
TG(18:1/18:1/16:0)	Upregulated				
TG(18:1/12:0/18:1) or	Upregulated				
TG(18:0/18:1/20:4)	Upregulated				
TG(16:0/22:5/18:1) or	Upregulated				
TG(16:0/18:0/18:1)	Upregulated				
TG(14:0/18:2/18:2)	Upregulated				
TG(14:0/18:1/18:1)	Upregulated				
TG(14:0/16:0/18:1)	Upregulated				
SM(d41:1)	Upregulated				
SM(d38:2)	Upregulated				
SM(d36:2)	Upregulated				
SM(d36:1)	Upregulated				
SM(d36:0)	Upregulated				
SM(d34:1)	Upregulated				

Supplementary table S1: Individual lipid alterations in SSD and related psychoses.

Lipid name	Change in levels	Involvement	Patient details	Biofluid	REF
SM(d18:2/14:0)	Upregulated				
SM(d18:1/24:0)	Upregulated				
SM(d34:2)	Upregulated				
PI(18:0/20:4)	Upregulated				
PC(O-22:2/22:3)	Upregulated				
PC(40:5)	Upregulated				
PC(40:4)	Upregulated				
PC(38:4)	Upregulated				
PC(38:2)	Upregulated				
LPC(20:3)	Upregulated				
LPC(16:1)	Upregulated				
Cer(d18:1/24:1)	Upregulated				
Cer(d18:1/24:0)	Upregulated				
CE(20:3)	Upregulated				
TG(48:0)	Positively associated with	12-month follow up vs baseline of FEP patients	Patients on various treatments (treatment type unspecified)	Plasma	Lamichhane, et al. (86)
TG(47:0)	Positively associated with				
TG(48:0)	Positively associated with				
TG(47:1)	Positively associated with				
TG(48:1)	Positively associated with				
TG(48:1)	Positively associated with				
TG(14:0/16:0/18:1)	Positively associated with				
TG(16:0/16:0/16:0)	Positively associated with				
TG(49:0)	Positively associated with				
TG(14:0/16:0/18:1)	Associated with BMI	12-month follow up vs baseline of CHR patients	Patients on various treatments (treatment type unspecified)	Plasma	
TG(14:0/18:1/18:1)	Associated with BMI				
TG(14:0/18:2/18:2)	Associated with BMI				
TG(14:0/18:2/18:2)	Associated with BMI				
TG(16:0/16:0/16:0)	Associated with BMI				
TG(16:0/18:0/18:1)	Associated with BMI				
TG(16:0/18:2/18:2)	Associated with BMI				
TG(16:0/18:2/18:3)	Associated with BMI				
TG(16:0/18:2/22:6)	Associated with BMI				
TG(16:0/18:2/22:6)	Associated with BMI				
TG(16:0/22:5/18:1) or	Associated with BMI				
TG(16:0/22:5/18:1) or	Associated with BMI				
TG(18:0/18:0/18:0)	Associated with BMI				
TG(18:0/18:1/20:4)	Associated with BMI				
TG(18:0/18:1/20:4)	Associated with BMI				
TG(18:1/12:0/18:1) or	Associated with BMI				
TG(18:1/18:1/16:0)	Associated with BMI				
TG(18:1/18:1/18:1)	Associated with BMI				
TG(18:1/18:1/22:6)	Associated with BMI				
TG(18:1/18:1/22:6)	Associated with BMI				
TG(18:1/18:2/18:2)	Associated with BMI				
TG(18:1/18:2/18:2)	Associated with BMI				
TG(18:2/18:1/16:0)	Associated with BMI				
TG(18:2/18:1/18:1)	Associated with BMI				
TG(18:2/18:1/18:1)	Associated with BMI				
TG(18:2/18:2/18:2) or	Associated with BMI				
TG(18:2/18:2/18:2) or	Associated with BMI				
TG(18:2/18:2/18:2) or	Associated with BMI				
TG(18:2/22:5/16:0)	Associated with BMI				
TG(18:2/22:5/16:0)	Associated with BMI				
TG(37:0)	Associated with BMI				
TG(45:0)	Associated with BMI				
AEA	Upregulated	Twins discordant for SCZ vs healthy controls	Mix of patients with and without cannabis substance abuse	Plasma	Koethe, et al. (87)
PEA	Upregulated				
2-AG	Downregulated	FEP patients vs. healthy controls	Drug naïve FEP patients	Serum	
AEA	Upregulated				
LEA	Upregulated				
OEA	Upregulated				
PEA	Upregulated				
PC aa C24:0	Upregulated				
PC aa C26:0	Downregulated				
PC aa C28:1	Downregulated				
PC aa C30:0	Downregulated				
PC aa C30:2	Downregulated				
PC aa C32:0	Downregulated				
PC aa C32:1	Downregulated				
PC aa C32:2	Downregulated				
PC aa C32:3	Downregulated				
PC aa C34:1	Downregulated				
PC aa C34:2	Downregulated				
PC aa C34:3	Downregulated				
PC aa C34:4	Downregulated				
PC aa C36:0	Upregulated				
PC aa C36:1	Downregulated				
PC aa C36:2	Downregulated				
PC aa C36:3	Downregulated				

Supplementary table S1: Individual lipid alterations in SSD and related psychoses.

Lipid name	Change in levels	Involvement	Patient details	Biofluid	REF
PC aa C36:4	Downregulated				
PC aa C36:5	Downregulated				
PC aa C36:6	Downregulated				
PC aa C38:0	Downregulated				
PC aa C38:1	Upregulated				
PC aa C38:3	Downregulated				
PC aa C38:4	Downregulated				
PC aa C38:5	Downregulated				
PC aa C38:6	Downregulated				
PC aa C40:1	Upregulated				
PC aa C40:2	Upregulated				
PC aa C40:3	Upregulated				
PC aa C40:4	Upregulated				
PC aa C40:5	Downregulated				
PC aa C40:6	Downregulated				
PC aa C42:0	Upregulated				
PC aa C42:1	Upregulated				
PC aa C42:2	Upregulated				
PC aa C42:4	Upregulated				
PC aa C42:5	Upregulated				
PC aa C42:6	Downregulated				Parksepp, et al. (88)
2-AG	Upregulated				
AEA	Downregulated				
LEA	Downregulated				
OEA	Downregulated				
PEA	Downregulated				
PC aa C24:0	Downregulated				
PC aa C26:0	Downregulated				
PC aa C28:1	Upregulated				
PC aa C30:0	Upregulated				
PC aa C30:2	Downregulated				
PC aa C32:1	Upregulated				
PC aa C32:2	Upregulated				
PC aa C32:3	Downregulated				
PC aa C34:1	Upregulated				
PC aa C34:2	Upregulated				
PC aa C34:3	Upregulated				
PC aa C34:4	Upregulated				
PC aa C36:0	Downregulated				
PC aa C36:1	Upregulated				
PC aa C36:2	Upregulated				
PC aa C36:3	Upregulated	Pre- vs. post- antipsychotics	FEP patients	Serum	
PC aa C36:4	Upregulated				
PC aa C36:5	Upregulated				
PC aa C36:6	Upregulated				
PC aa C38:0	Downregulated				
PC aa C38:1	Downregulated				
PC aa C38:3	Upregulated				
PC aa C38:4	Upregulated				
PC aa C38:5	Upregulated				
PC aa C38:6	Upregulated				
PC aa C40:1	Downregulated				
PC aa C40:2	Downregulated				
PC aa C40:3	Downregulated				
PC aa C40:4	Downregulated				
PC aa C40:5	Upregulated				
PC aa C40:6	Upregulated				
PC aa C42:0	Downregulated				
PC aa C42:1	Downregulated				
PC aa C42:2	Downregulated				
PC aa C42:4	Downregulated				
PC aa C42:5	Downregulated				
PC aa C42:6	Downregulated				
CerP(m/z 668.4981)	Downregulated in poor				
DG(m/z 613.4787)	Downregulated in poor				
PA(m/z 645.4850)	Downregulated in poor				
PC(m/z 508.3747)	Downregulated in poor				
PC(m/z 868.5270)	Downregulated in poor				
PC(m/z 870.5429)	Downregulated in poor				
PG(m/z 831.5516)	Downregulated in poor				
PS(m/z 840.5693)	Downregulated in poor				
PS(m/z 812.5225)	Downregulated in poor				
PS(m/z 824.5750)	Downregulated in poor				
PS(m/z 794.528)	Downregulated in poor				
SM(m/z 837.6211)	Downregulated in poor				
TG(m/z 907.7158)	Downregulated in poor				
PC(m/z 874.5758)	Downregulated in good				
PC(m/z 808.5834)	Downregulated in good				
PC(m/z 880.7094)	Downregulated in good	Treatment response	Drug naïve/drug free (6 wks off) SCZ patients	Plasma	de Almeida, et al. (91)

Supplementary table S1: Individual lipid alterations in SSD and related psychoses.

Lipid name	Change in levels	Involvement	Patient details	Biofluid	REF
PC(m/z 800.6515)	Upregulated in good		SCZ patients		(91)
PC(m/z 832.5791)	Upregulated in good				
PE(m/z 692.5198)	Upregulated in good				
PI-Cer(m/z 892.5660)	Downregulated in good				
PS(*m/z* 813.5488n)	Downregulated in poor				
PA(*m/z* 825.5735)	Downregulated in poor				
PS(*m/z* 771.5408n)	Downregulated in poor				
PA(*m/z* 703.5610)	Upregulated in good				
PC(*m/z* 832.5791)	Upregulated in good				
PG(*m/z* 1578.1972)	Upregulated in good				
PG(*m/z* 1578.1972)	Upregulated in poor				
PS(m/z 812.5225)	Downregulated in poor				
GlcCer(m/z 792.5780)	Downregulated in poor				
PG(m/z 749.5304)	Downregulated in poor				
PI(m/z 909.5424)	Downregulated in poor				
CE 16:1	Upregulated	SCZ patients vs. healthy controls	Patients before antipsychotic treatment	Plasma	Yan, et al. (92)
CE 18:1	Upregulated				
CE 18:3	Upregulated				
CE 20:3	Upregulated				
CE 20:4	Upregulated				
CE 22:6	Upregulated				
LPC 14:0	Downregulated				
LPC 18:0	Downregulated				
LPC 18:2	Downregulated				
LPC 20:0	Downregulated				
LPC 20:2	Downregulated				
LPC 22:0	Downregulated				
LPC 22:4	Upregulated				
LPC 22:6	Downregulated				
LPE 16:0	Downregulated				
LPE 18:1	Downregulated				
PC(18:2/18:2)	Downregulated				
PC(18:2/18:3)	Downregulated				
p-PC(P-14:0/2:0)	Downregulated				
p-PC(P-14:0/20:2)	Downregulated				
p-PC(P-16:0/18:2)	Downregulated				
p-PC(P-16:0/20:4)	Downregulated				
p-PC(P-18:0/20:4)	Downregulated				
p-PC(P-20:0/18:5)	Downregulated				
p-PE(P-16:0/18:1)	Downregulated				
p-PE(P-16:0/18:2)	Downregulated				
p-PE(P-18:0/18:1)	Downregulated				
p-PE(P-18:0/18:2)	Downregulated				
p-PE(P-18:0/18:3)	Downregulated				
p-PE(P-16:0/20:4)	Downregulated				
p-PE(P-20:0/18:2)	Downregulated				
p-PE(P-18:0/20:3)	Downregulated				
p-PE(P-18:0/20:4)	Downregulated				
p-PE(P-16:0/22:5)	Downregulated				
p-PE(P-16:0/22:6)	Downregulated				
p-PE(P-18:0/22:4)	Downregulated				
p-PE(P-18:0/22:6)	Downregulated				
SM(d14:2/26:2)	Upregulated				
TG(16:0/16:0/18:1)	Upregulated				
TG(16:0/18:1/18:1)	Upregulated				
TG(16:0/20:1/20:3)	Upregulated				
TG(16:0/18:1/22:4)	Upregulated				
TG(18:1/18:1/20:4)	Upregulated				
TG(18:1/18:2/20:4)	Upregulated				
TG(18:2/18:2/22:6)	Upregulated				
TG(18:1/20:4/20:4)	Upregulated				
CE 16:0	Downregulated				
CE 18:1	Downregulated				
CE 18:2	Downregulated				
CE 20:3	Downregulated				
CE 20:4	Downregulated				
CE 20:5	Downregulated				
CE 22:6	Downregulated				
Cer(d18:0/22:0)	Downregulated				
Cer(d18:0/24:0)	Downregulated				
FA 16:0	Downregulated				
FA 16:1	Downregulated				
FA 18:0	Downregulated				
FA 18:1	Downregulated				

Supplementary table S1: Individual lipid alterations in SSD and related psychoses.

Lipid name	Change in levels	Involvement	Patient details	Biofluid	REF
FA 20:1	Downregulated	Pre- vs. post- antipsychotics	Haloperidol, Clozapine, Quetiapine, Risperidone, Olanzapine	Plasma	
FA 20:2	Downregulated				
FA 20:4	Downregulated				
FA 22:4	Downregulated				
FA 22:6	Downregulated				
GlcCer(d18:1/22:0)	Downregulated				
GlcCer(d18:1/24:0)	Downregulated				
GlcCer(d18:1/24:1)	Downregulated				
LPC 18:2	Downregulated				
LPC 22:6	Downregulated				
PC(16:0/16:0)	Downregulated				
PC(16:0/20:4)	Downregulated				
PC(18:0/20:4)	Downregulated				
PC(16:0/22:6)	Downregulated				
PC(18:0/22:6)	Downregulated				
p-PC(P-14:0/22:1)	Downregulated				
p-PC(P-14:0/22:3)	Downregulated				
p-PC(P-16:0/22:3)	Downregulated				
p-PC(P-14:0/24:4)	Downregulated				
p-PC(P-20:0/18:5)	Downregulated				
p-PC(P-16:0/22:6)	Downregulated				
p-PC(P-18:0/22:3)	Downregulated				
p-PC(P-14:0/26:4)	Downregulated				
p-PC(P-18:0/22:6)	Downregulated				
p-PC(P-20:0/22:3)	Downregulated				
p-PC(P-16:0/26:4)	Downregulated				
p-PC(P-22:0/20:4)	Downregulated				
p-PC(P-20:0/22:5)	Downregulated				
p-PC(P-18:0/26:4)	Downregulated				
SM(d14:1/26:2)	Downregulated				
SM(d14:1/30:2)	Downregulated				
TG(16:0/18:2/22:6)	Downregulated				
TG(18:1/18:1/22:5)	Downregulated				
LPC(16:1)	Upregulated	Patients with psychotic experiences vs. controls	Children at 12 years with psychotic experiences	Plasma	Madrid-Gambin, et al. (~~98~~)
LPC(18:1)	Upregulated				
LPC(18:2)	Upregulated				
LPC(20:3)	Upregulated				
PC(30:0)	Upregulated				
PC(32:0)	Upregulated				
PC(32:1)	Upregulated				
PC(34:2)	Upregulated				
PC(36:1)	Upregulated				
PC(36:2)	Upregulated				
PC(36:3)	Upregulated				
PC(36:4)	Upregulated				
PC(38:2)	Upregulated				
PC(38:3)	Upregulated				
PC(O-38:6)	Upregulated				
PC(34:1)	Upregulated				

The basic structure of lipids identified by de Almeida, et al. (86) was not determined.

List of abbreviations: AA, Arachidonic acid; AEA, Anandamide; BCFA, Branched chain fatty acids; BMI, body-mass index; Cer, Ceramide; CerP, Ceramide phosphate; CE, Cholesterol ester; CHR, Clinical high risk for psychosis; DG, Diglyceride; DHA, Docosahexanoic acid; DHET, di-hydroxy-eicosatrienoic acid; DiHOME, dihydroxyoctadecenoic acid; EA, Ethanolamide; eCB, Endocannabinoid; EPA, Eicosapentanoic acid; FA, Fatty acid; FEP, First-episode psychosis; FFA, Free fatty acid; HDoHE, hydroxyl-docasahexaenoic acid; HETE, hydroxy-eicosadienoic acid; HODE, hydroxy-octadecadienoic acid; HpETE, hydroperoxy-eicosatetraenoic acid; LEA, Linoleoylethanolamide; LPC, Lysophosphatidylcholine; GlcCer, Glucosylceramide; KEDE, oxo-
